# Utilizing a reductionist model to study host-microbe interactions in intestinal inflammation

**DOI:** 10.1186/s40168-021-01161-3

**Published:** 2021-11-03

**Authors:** Amy M. Tsou, Jeremy A. Goettel, Bin Bao, Amlan Biswas, Yu Hui Kang, Naresh S. Redhu, Kaiyue Peng, Gregory G. Putzel, Jeffrey Saltzman, Ryan Kelly, Jordan Gringauz, Jared Barends, Mai Hatazaki, Sandra M. Frei, Rohini Emani, Ying Huang, Zeli Shen, James G. Fox, Jonathan N. Glickman, Bruce H. Horwitz, Scott B. Snapper

**Affiliations:** 1 Division of Gastroenterology, Hepatology and Nutrition, Boston Children’s Hospital, Boston, MA USA; 2 Harvard Medical School, Boston, MA USA; 3 Jill Roberts Institute for Research in Inflammatory Bowel Disease, Weill Cornell Medical College, New York, NY USA; 4 Division of Pediatric Gastroenterology and Nutrition, Weill Cornell Medical College, New York, NY USA; 5 Division of Gastroenterology, Hepatology, and Nutrition, Department of Medicine, Vanderbilt University Medical Center, Nashville, TN USA; 6 Department of Gastroenterology, Children’s Hospital of Fudan University, Shanghai, China; 7 Division of Comparative Medicine, Massachusetts Institute of Technology, Cambridge, MA USA; 8 Department of Pathology, Beth Israel Deaconess Medical Center, Boston, MA USA; 9 Division of Emergency Medicine, Boston Children’s Hospital, Boston, MA USA; 10 Division of Gastroenterology, Brigham and Women’s Hospital, Boston, MA USA

**Keywords:** Intestinal inflammation, Wiskott-Aldrich syndrome, Immune dysregulation, Gut microbiota, Defined consortium, Pathobiont

## Abstract

**Background:**

The gut microbiome is altered in patients with inflammatory bowel disease, yet how these alterations contribute to intestinal inflammation is poorly understood. Murine models have demonstrated the importance of the microbiome in colitis since colitis fails to develop in many genetically susceptible animal models when re-derived into germ-free environments. We have previously shown that Wiskott-Aldrich syndrome protein (WASP)-deficient mice (*Was*^*−/−*^) develop spontaneous colitis, similar to human patients with loss-of-function mutations in *WAS*. Furthermore, we showed that the development of colitis in *Was*^*−/−*^ mice is *Helicobacter* dependent. Here, we utilized a reductionist model coupled with multi-omics approaches to study the role of host-microbe interactions in intestinal inflammation.

**Results:**

*Was*^*−/−*^ mice colonized with both altered Schaedler flora (ASF) and *Helicobacter* developed colitis, while those colonized with either ASF or *Helicobacter* alone did not. In *Was*^*−/−*^ mice, *Helicobacter* relative abundance was positively correlated with fecal lipocalin-2 (LCN2), a marker of intestinal inflammation. In contrast, WT mice colonized with ASF and *Helicobacter* were free of inflammation and strikingly, *Helicobacter* relative abundance was negatively correlated with LCN2. In *Was*^*−/−*^ colons, bacteria breach the mucus layer, and the mucosal relative abundance of ASF457 *Mucispirillum schaedleri* was positively correlated with fecal LCN2. Meta-transcriptomic analyses revealed that ASF457 had higher expression of genes predicted to enhance fitness and immunogenicity in *Was*^*−/−*^ compared to WT mice. In contrast, ASF519 *Parabacteroides goldsteinii*’s relative abundance was negatively correlated with LCN2 in *Was*^*−/−*^ mice, and transcriptional analyses showed lower expression of genes predicted to facilitate stress adaptation by ASF519 in *Was*^*−/−*^compared to WT mice.

**Conclusions:**

These studies indicate that the effect of a microbe on the immune system can be context dependent, with the same bacteria eliciting a tolerogenic response under homeostatic conditions but promoting inflammation in immune-dysregulated hosts. Furthermore, in inflamed environments, some bacteria up-regulate genes that enhance their fitness and immunogenicity, while other bacteria are less able to adapt and decrease in abundance. These findings highlight the importance of studying host-microbe interactions in different contexts and considering how the transcriptional profile and fitness of bacteria may change in different hosts when developing microbiota-based therapeutics.

Video abstract

**Supplementary Information:**

The online version contains supplementary material available at 10.1186/s40168-021-01161-3.

## Introduction

Inflammatory bowel diseases (IBD) are a group of heterogeneous chronic inflammatory disorders that affect the gastrointestinal tract. The etiology of IBD is complex and multifactorial, with genetic, immunological, environmental, and microbial factors all contributing. Genome-wide association studies have identified more than 240 loci associated with a risk of IBD, and many of these loci implicate pathways involved in immunological responses to bacteria [1–3]. The intestines, particularly the colon, are inhabited by a high density of bacteria, and many lines of evidence suggest that these microbes, known as the intestinal microbiota, play an important role in IBD. For example, the composition of the intestinal microbiota is altered, or dysbiotic, in patients with IBD compared to healthy controls, although the specific differences are highly variable among studies [4]. Furthermore, antibiotics, fecal stream diversion, and fecal microbiota transplants have all been shown to be somewhat efficacious in the treatment of IBD, although our ability to draw definitive conclusions about their efficacy has been hindered by heterogeneous study designs [5–10]. In order to improve upon microbial-based therapeutics for IBD, we need to develop a better understanding of how gut microbes initiate and modulate intestinal inflammation and how inflammation shapes the gut microbiota.

Human microbiome studies can be difficult to interpret due to variation in genetics, diet, and other environmental factors that shape microbial composition aside from the examined disease state. It is also challenging to obtain samples from patients prior to the onset of disease or longitudinally thereafter, limiting the conclusions that can be drawn from these studies. In contrast, murine models of disease lack much of this confounding complexity, and their microbiota can be user defined and longitudinally sampled. Murine studies have been instrumental in establishing the importance of the gut microbiota in initiating colitis, since colitis fails to develop in nearly all murine models of IBD when mice are re-derived in germ-free environments [11, 12]. In addition, the colitis phenotype of certain murine models is communicable by transfer of the gut microbiota [13, 14]. Murine models have also facilitated the identification of immunomodulatory molecules produced by gut bacteria such as short-chain fatty acids [15–17] and capsular polysaccharide A [18]. However, the design of rigorous, well-controlled microbiome studies in mice is often limited by the large number of bacterial species involved and inter-individual variation in gut microbial communities. To address this, we have developed a reductionist model using a simplified and defined microbial consortium in a genetically susceptible host.

Similar to humans, mice lacking the Wiskott-Aldrich syndrome protein (WASP) (*Was*^*−/−*^ mice) exhibit immune cell defects leading to gut inflammation [19]. Furthermore, colitis in *Was*^*−/−*^ mice is microbiota-dependent. WASP is a hematopoietic-specific protein, encoded on the X chromosome that regulates Arp2/3-dependent actin polymerization, and mice and humans deficient in WASP exhibit defects in both their adaptive and innate immune systems [20, 21]. WASP-deficient patients develop a primary immunodeficiency characterized by recurrent infections, eczema, and thrombocytopenia, and approximately 10% develop an IBD-like colitis [22]. *Was*^*−/−*^ mice on the 129SvEv background housed under specific pathogen-free (SPF) conditions containing *Helicobacter* species reliably develop a spontaneous lymphocyte-dependent colitis [23, 24]. SPF mice contain a complex microbial community that is free of particular disease-causing microbes. When *Was*^*−/−*^ mice are re-derived in SPF conditions free of *Helicobacter*, they no longer develop spontaneous intestinal inflammation. Following colonization of re-derived SPF *Was*^*−/−*^ mice with *Helicobacter bilis*, they develop typhlitis and colitis, with a subset exhibiting dysplasia and even colon carcinoma [24]. In this study, we show that mono-colonization of *Was*^*−/−*^ mice with *H. bilis* is not sufficient to induce intestinal inflammation, indicating that *H. bilis* requires the presence of other bacteria to elicit colitis in *Was*^*−/−*^ mice. Furthermore, we replace the complex and variable SPF flora with a defined commensal community, the altered Schaedler flora (ASF) [25–27], to study how interactions between a pathobiont, the commensal flora, and an immune-dysregulated host result in the initiation and perpetuation of intestinal inflammation.

## Results

### WASP deficiency is associated with altered fecal microbial composition and outgrowth of pathobionts

**Fig. 1.**
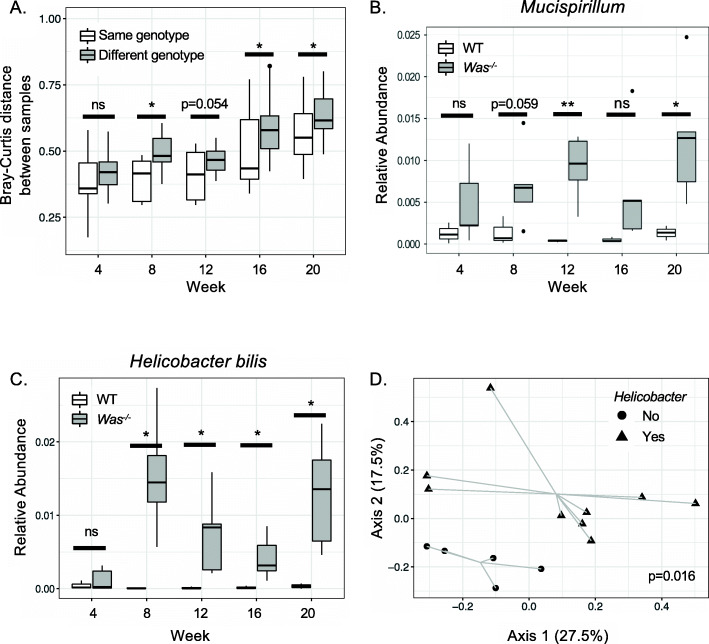
WASP deficiency results in altered composition of the fecal microbiota. **A–C** Fecal microbial composition of *Was*^*−/−*^ (*n* = 5) and WT (*n* = 3) mice raised under SPF conditions with weekly bedding exchanges was analyzed monthly between 4 and 20 weeks of age by 16S rRNA gene sequencing. **A** Compositional dissimilarity between mice of the same genotype compared to mice of different genotypes was assessed by Bray-Curtis distance. Relative abundances of *Mucispirillum* (**B**) and *H. bilis* (**C**) were determined for each genotype at each timepoint. **D** SPF *Was*^*−/−*^ mice were re-derived in a *Helicobacter* species free environment (*n* = 14), and a subset was infected with *H. bilis* (*n* = 9)*.* Fecal microbial composition was assessed at 7–9 months post infection. Principal coordinate analysis of Bray-Curtis distances was performed after *H. bilis* sequences were removed from the dataset. Statistics performed using PERMANOVA (**A** and **D**) and Student’s *t*-test (**B** and **C**). *p < 0.05, **p < 0.01

We first sought to determine whether gut microbial composition in *Was*^*−/−*^ mice differs from wild-type (WT) mice and if so, at what age their microbiota begin to diverge. Stool samples were collected longitudinally from *Was*^*−/−*^ and WT mice raised under SPF conditions at 4, 8, 12, 16, and 20 weeks of age. Microbial profiling by 16S rRNA sequencing revealed that the microbiota of WT and *Was*^*−/−*^ mice were similar at 4 weeks of age and began to differ by 8 weeks of age, as measured by Bray-Curtis distance (Fig. 1A). Analysis at the phylum level revealed that only the relative abundance of *Deferribacteres* was consistently and significantly different between genotypes after correcting for multiple taxa comparisons (Fig. S1A,B). Further analysis revealed that the genus *Mucispirillum* accounted for essentially 100% of the *Deferribacteres* reads, and the *Mucispirillum* relative abundance was higher in *Was*^*−/−*^ compared to WT mice after week 8 (Fig. 1B). This finding is consistent with prior studies demonstrating outgrowth of *Mucispirillum* under inflammatory conditions and a role for *Mucispirillum schaedleri* as a pathobiont that triggers inflammation in an immune-deficient host [28, 29].

Given our prior study revealing a critical role for *H. bilis* in colitis associated with WASP deficiency [24], we examined the relative abundance of this species and found that it was consistently higher in *Was*^*−/−*^ compared to WT mice at 8 weeks of age and thereafter (Fig. 1C). Finally, we asked whether the presence of *H. bilis* leads to changes in other members of the intestinal microbial community. 16S rRNA sequencing of feces collected from *Was*^*−/−*^ mice re-derived in a *Helicobacter-*free environment and a subset of those that were re-colonized with *H. bilis* showed significant differences in microbial composition, even after removal of sequences attributable to *H. bilis* (Fig. 1D)*.* Taken together, these findings indicate that WASP deficiency is associated with changes in fecal microbial composition, including expansion of pathobionts such as *H. bilis* and *Mucispirillum*, and the presence of *H. bilis* drives alterations of other members of the microbial community.

### Development of a reductionist model to study the role of the gut microbiota in intestinal inflammation

**Fig. 2:**
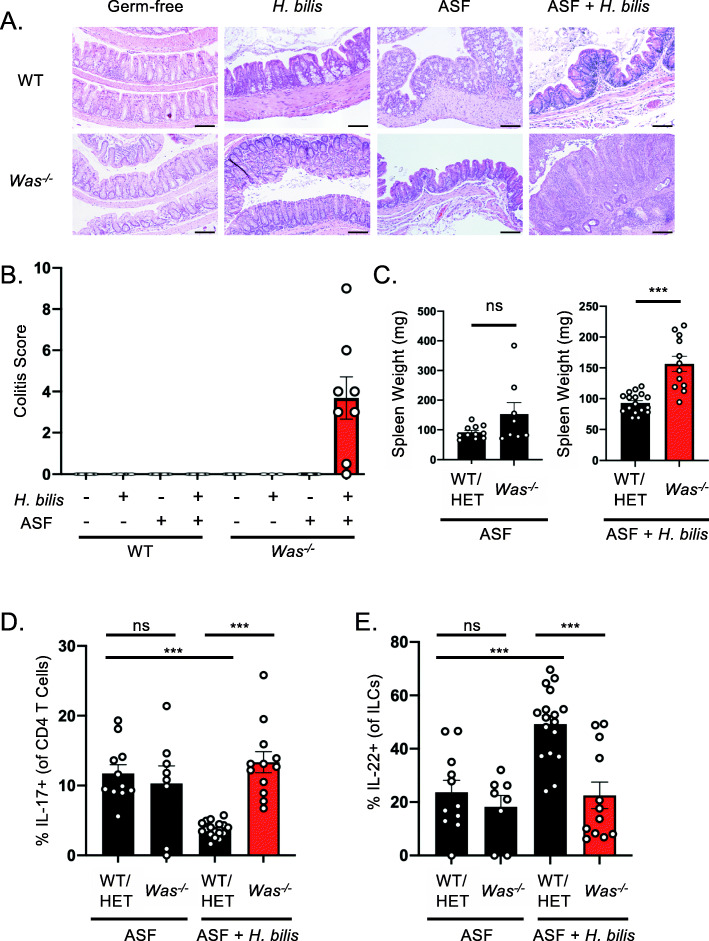
Development of intestinal inflammation requires both the pathobiont, *H. bilis*, as well as commensal bacteria in the context of host immune dysregulation. **A**, **B** WT and *Was*^*−/−*^ mice were colonized with the indicated gut microbial communities. **A** Representative H&E-stained formalin-fixed paraffin-embedded proximal colon sections at 20 weeks after colonization. 20× magnification, scale bars = 100μm. **B** Quantitative histological colitis scores at 20 weeks after colonization (*n* = 5,4,5,5,6,3,8,8). **C–E** Germ-free WT/HET (n=28) and *Was*^*−/−*^ (*n* = 21) mice were colonized with the ASF community and a subset (WT/HET (*n* = 17) and *Was*^*−/−*^ (*n* = 13)) were gavaged with *H. bilis*. **C** Spleen weights were measured 20 weeks after gavage with *H. bilis.* Colonic lamina propria lymphocytes were isolated 20 weeks after infection with *H. bilis*, and percentages of IL17-A^+^ CD4 T cells (**D**) and IL-22^+^ ILC3s (**E**) were determined by flow cytometry. Statistics performed using Student’s *t*-test. *p < 0.05, ***p < 0.001

Next, we sought to determine whether other gut bacteria might contribute to the intestinal inflammation observed in *Was*^*−/−*^ mice colonized with *H. bilis*. The complexity and variability of SPF microbiota make rigorous mechanistic studies challenging. Therefore, we sought to establish a reductionist gnotobiotic model using ASF, which is a defined microbial community comprised of 8 members [25–27]. Historically, ASF was developed to colonize germ-free mice with a standardized microbiota, and it has been shown to be more functionally representative of wild microbiomes compared to random consortia of similar or larger size [25, 26, 30]. Notably, ASF does contain *M. schaedleri*, also known as ASF457, which as described above, has been shown to be a pathobiont in certain contexts [29]. Colonization of *Was*^*−/−*^ mice with ASF alone did not induce spontaneous colitis (Fig. 2A, B). Mono-colonization with *H. bilis* also did not induce inflammation in *Was*^*−/−*^ mice, but *Was*^*−/−*^ mice colonized with both ASF and *H. bilis* developed inflammation (Fig. 2A, B), similar to our prior work demonstrating colitis development in SPF *Was*^*−/−*^ mice colonized with *H. bilis* [24]. Colonization of WT mice with both ASF and *H. bilis* was not sufficient to induce colitis (Fig. 2A, B). These findings indicate that spontaneous colitis in *Was*^*−/−*^ mice requires the presence of both a pathobiont and other bacteria in the context of an immune-dysregulated host.

### The effect of *H. bilis* on colonic lamina propria immune cells is context dependent

To study the temporal relationship between the onset of inflammation and changes in the intestinal microbiota, we designed an experiment in which germ-free WT, *Was*^*+/−*^ (HET), and *Was*^*−/−*^ mice were colonized with ASF, with a cohort subsequently infected with *H. bilis* (Fig. S2A). To transfer the ASF community to germ-free mice, donor ASF-colonized mice obtained from Taconic Biosciences were co-housed with germ-free mice for 2 months (females), or stool-soiled bedding was transferred from ASF donor cages to recipient cages weekly for 2 months (males). 16S rRNA sequencing confirmed successful colonization of the recipient mice with ASF (Fig. S2B). ASF360 *Lactobacillus intestinalis* was not detected in donor or recipient fecal samples, which is consistent with prior reports [31].

As expected, *Was*^*−/−*^ but not WT mice colonized with ASF and *H. bilis* developed histologic evidence of intestinal inflammation. Inflammation was observed throughout the cecum and colon (Fig. S2C). Spleen weights were also significantly increased in the *Was*^*−/−*^ mice colonized with ASF and *H. bilis* (Fig. 2C)*.* Analysis of colonic lamina propria lymphocytes revealed an increased percentage of IL-17A^+^ CD4 T cells among total CD4 T cells in colitic *Was*^*−/−*^ mice that were colonized with ASF and *H. bilis* as compared to WT mice colonized with the same microbiota (Figs. 2D and S2D)*.* Notably, addition of *H. bilis* to WT mice colonized with ASF led to a significant decrease in the percentage of IL-17A+ CD4 T cells compared to WT mice colonized with ASF alone (Fig. 2D), These data are consistent with recent studies showing that *Helicobacter* species induce regulatory T cells (T_reg_) that restrain pro-inflammatory T helper 17 (T_H_17) cells during homeostasis while the same bacteria promote expansion of colitogenic T_H_17 cells during inflammation [32, 33]. Furthermore, *Helicobacter hepaticus* has been shown to activate an anti-inflammatory program in macrophages [34].

Group 3 innate lymphoid cells (ILC3s) help maintain intestinal homeostasis and ameliorate inflammation via production of IL-22 [35–37]. *Helicobacter* species have been shown to negatively regulate the proliferation of ILC3s in an immune-deficient background, and consistent with this, we observed a lower percentage of ILC3s among total ILCs in the colonic lamina propria of *Was*^*−/−*^ mice that were colonized with ASF and *H. bilis* as compared to WT mice colonized with the same microbiota [38] (Figs. 2E and S2E). However, *H. bilis*’s effect on ILC3s is context dependent, and in uninflamed WT mice, *H. bilis* colonization resulted in an increase in the percentage of ILC3s (Fig. 2E). These findings highlight that the effect of a given microorganism on the immune compartment can vary greatly depending on whether they are in a homeostatic or immune dysregulated environment, and specifically in the case of *H. bilis*, it induces a tolerogenic response during homeostasis but promotes inflammation in immune dysregulated hosts.

### Fecal microbial composition is correlated with degree of inflammation

**Fig. 3:**
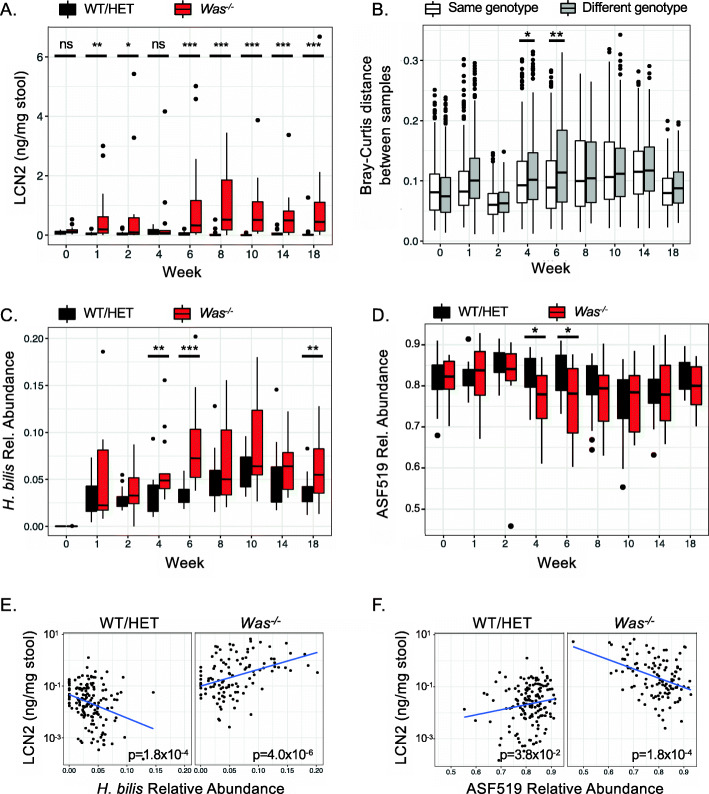
Correlation between the relative abundance of the pathobiont *H. bilis* and the degree of intestinal inflammation depends upon the host genotype. Germ-free WT/HET (n=17) and *Was*^*−/−*^ (*n* = 13) mice were colonized with the ASF community and then gavaged with *H. bilis*. **A** Development of intestinal inflammation was monitored by measuring fecal LCN2 serially. Fecal microbial composition was assessed longitudinally by 16S rRNA sequencing. **B** Compositional dissimilarity between mice of the same genotype compared to mice of different genotypes as measured by Bray-Curtis distance. Relative abundance of *H. bilis* (**C**) and ASF519 *P. goldsteinii* (**D**) over time by genotype*.* Correlations between log-transformed fecal LCN2 and relative abundances of *H. bilis* (**E**) and ASF519 *P. goldsteinii* (**F**) for all timepoints. Tests for linear dependence of log-transformed LCN2 on the relative abundance of each bacterial species was done using a linear mixed-effects model, taking into account a mouse-specific random effect (E and F). Wilcoxon’s rank-sum test was used for (**A**), (**C**), and (**D**) and PERMANOVA for (**B**). *p < 0.05, **p < 0.01, ***p < 0.001

We next performed longitudinal fecal sample collection from these mice to elucidate the temporal relationship between the onset of inflammation and changes in microbial composition. Intestinal inflammation developed in *Was*^*−/−*^ mice within a week after *H. bilis* gavage, as measured by fecal lipocalin-2 (LCN2) (Fig. 3A). *Was*^*−/−*^ mice were able to control the acute inflammation by week 4 but then proceeded to develop persistent inflammation by week 6 (Fig. 3A). As expected, WT mice colonized with ASF and *H. bilis* (Fig. 3A) and WT or *Was*^*−/−*^ mice colonized with only ASF (Fig. S3A) did not show evidence of significant inflammation at any time during the experiment. *H. bilis* was not detected by 16S rRNA sequencing in stool from mice that were only colonized with ASF (Fig. S3B). We measured overall differences in microbial composition between WT and *Was*^*−/−*^ mice colonized with ASF and *H. bilis* and observed significant differences during weeks 4 and 6, which was just prior to and at the beginning of establishment of persistent inflammation in *Was*^*−/−*^ mice (Fig. 3A, B). The differences were primarily due to increased *H. bilis* and decreased ASF519 *Parabacteroides goldsteinii* relative abundances in *Was*^*−/−*^ mice (Fig. 3C, D). These differences were not due to cage effect, as there were multiple cages of each genotype that were separately housed, and the subset of WT/HET and *Was*^*−/−*^ mice that were co-housed still showed differences in *H. bilis* relative abundance (Fig. S3C).

Next, we assessed whether the fecal relative abundances of *H. bilis* and ASF519 *P. goldsteinii* correlated with the degree of inflammation using a linear mixed-effects model on data from all timepoints, taking into account a mouse-specific random effect. We found that *H. bilis* relative abundance was positively correlated with LCN2 in *Was*^*−/−*^ mice (Fig. 3E), consistent with its role as a pathobiont. Even though feces from WT/HET mice had low LCN2 levels overall, we were still able to detect a highly significant negative correlation between *H. bilis* relative abundance and LCN2 in WT/HET mice. These data mirror the context-dependent effects of *H. bilis* on T_H_17 cells (Fig. 2D). We also observed a negative correlation between ASF519 *P. goldsteinii* fecal relative abundance and LCN2, indicating a possible protective role for this organism (Fig. 3F), as suggested previously [39]. Analyses of the remaining ASF members did not reveal any strongly significant correlations between their fecal relative abundances and LCN2 levels (Fig. S3D-I).

### Mucosal-associated ASF457 *M. schaedleri* abundance is correlated with severity of inflammation

**Fig. 4:**
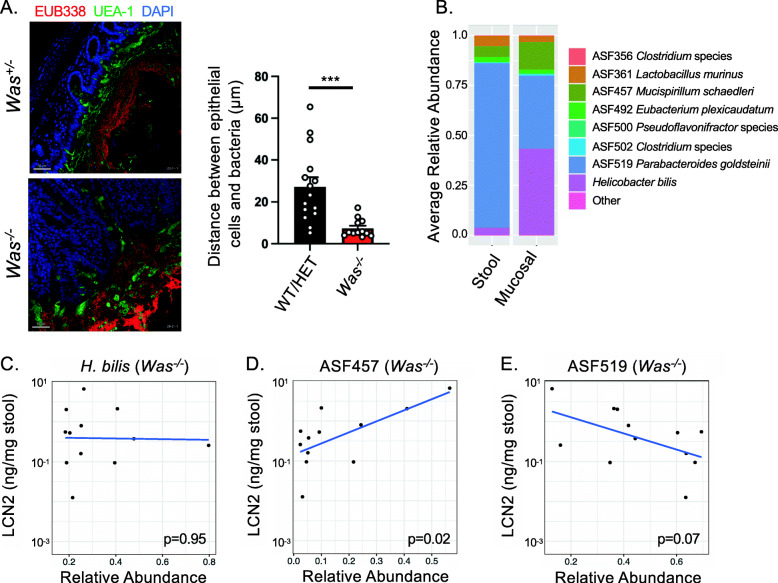
Colitis in WASP deficiency is associated with breach of the mucus barrier by the intestinal microbiota, and severity of colitis is correlated with the mucosal abundance of ASF457 *M. schaedleri*. Germ free WT/HET (n=17) and *Was*^*−/−*^ (*n* = 13) mice were colonized with the ASF community and then gavaged with *H. bilis*. The mucosal-associated microbiota was assessed at 20 weeks post infection. **A** Proximal colon sections from representative *Was*^*+/−*^ *and Was*^*−/−*^ mice stained with a universal bacterial probe (EUB338, red), for mucus using the lectin UEA-1 (green), and with DAPI (blue). Distances between epithelial cells and bacteria were quantified for 3 randomly chosen fields of view for each of 5 WT/HET and 4 *Was*^*−/−*^ mice. Each data point represents the average of 10 measurements taken across a field of view. Data shown as mean +/*−* S.E.M. Statistics done by Welch’s *t*-test. *** *p* < 0.001*.* **B** Comparison between fecal and mucosal-associated microbiota composition based on 16S rRNA sequencing. Pearson’s correlations between log-transformed fecal LCN2 and mucosal relative abundances of *H. bilis* (**C**), ASF457 *M. schaedleri* (**D**), and ASF519 *P. goldsteinii* (**E**) in *Was*^*−/−*^ mice

While stool sampling allows for convenient serial assessment of luminal bacteria, mucosal-associated bacteria are different in composition and are likely to be of particular relevance to intestinal inflammation given their close proximity to the host [40, 41]. Furthermore, in murine models of intestinal inflammation and human IBD, bacteria are able to penetrate the inner mucus layer, which usually separates bacteria from epithelial cells under homeostatic conditions [42]. Consistent with this, we observed that in colitic *Was*^*−/−*^ mice colonized with ASF and *H. bilis*, bacteria are able to penetrate the mucus layer and contact the host epithelium (Fig. 4A). When we profiled the composition of the mucosal-associated microbiota by 16S rRNA sequencing at 20 weeks after *H. bilis* infection, we found that *H. bilis* and ASF457 *M. schaedleri* are present at higher relative abundance than in the stool (Fig. 4B)*.* Both of these organisms are known to be mucus-dwelling [43, 44]. We then examined whether the mucosal relative abundances of each of the 3 most abundant bacteria in the mucosal samples correlated with fecal LCN2. We found no correlation for *H. bilis*, but mucosal ASF457 *M. schaedleri* correlated positively, and ASF519 *P. goldsteinii* trended towards a negative correlation with LCN2 in colitic *Was*^*−/−*^ mice colonized with ASF and *H. bilis* (Fig. 4C–E). In WT/HET mice, correlations between the mucosal abundances of each of these 3 bacteria and LCN2 trended in the same direction as in *Was*^*−/−*^ mice, but none of these correlations reached statistical significance (Fig. S4A-C). For the remaining ASF members, none of their mucosal abundances correlated with LCN2 in either genotype (Fig. S4D-H).

### WASP deficiency is associated with changes in bacterial gene expression

**Fig. 5:**
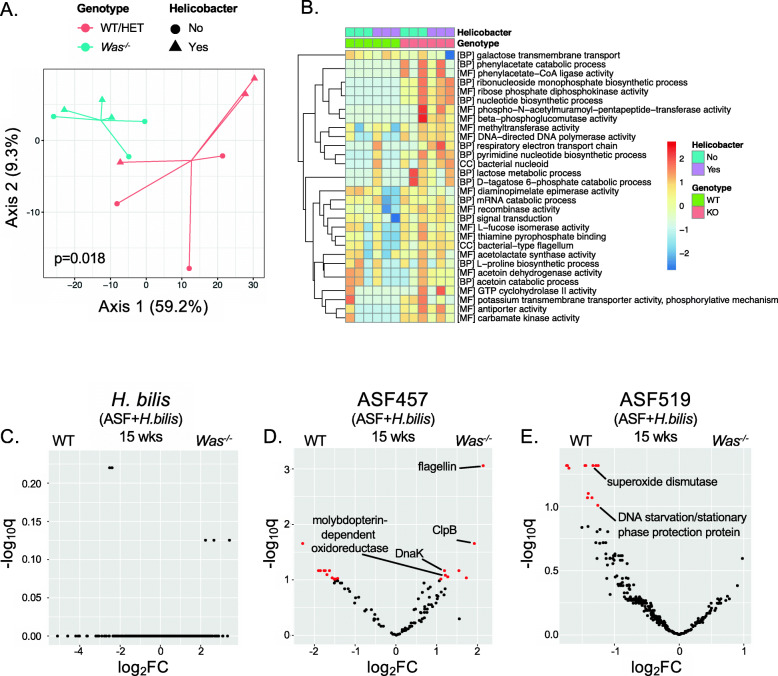
Colitis in WASP deficiency results in microbial transcriptional changes associated with stress adaptation and immunogenicity. Germ-free WT/HET and *Was*^*−/−*^ mice were colonized with the ASF community and a subset were gavaged with *H. bilis*. Feces from 3 mice of each genotype/microbiota combination (WT/HET or *Was*^*−/−*^ with or without *H. bilis*) at 15 weeks after *H. bilis* gavage were subjected to bacterial metatranscriptomic sequencing. **A** Principal component analysis of high variance Gene Ontology (GO) terms for all samples. Statistics calculated by PERMANOVA comparing WT/HET with *Was*^*−/−*^ samples. **B** Heatmap of high variance GO terms. Color scale indicates row-wise *z*-scores of log-transformed CPM values. BP, biological process: CC, cellular component, MF, molecular function. **C–E** RNA-seq volcano plots showing differential gene expression of *H. bilis* (**C**), ASF457 *M. schaedleri* (**D**), and ASF519 *P. goldsteinii* (**E**) in *Was*^*−/−*^ (positive log_2_(fold change (FC)) compared to WT/HET (negative log_2_(FC)) mice colonized with ASF and *H. bilis*. *Y*-axis shows *p*-values corrected for multiple comparison. Red dots represent genes that are significantly differentially expressed as determined by the DESeq2 Wald test

Assessment of microbial composition can provide important insights into how the microbiota respond to and contribute to intestinal inflammation, but these analyses do not consider the fact that bacteria have sophisticated sensory and signal transduction machinery that allows them to alter their transcriptional state in response to environmental cues. Therefore, even identical bacteria when placed in two different environments may behave very differently, and in doing so influence their host in diverse ways. To elucidate how WASP deficiency and/or inflammation may affect bacterial gene expression, we obtained stool samples from 3 mice of each of the 4 cohorts (WT/HET or *Was*^*−/−*^, ASF with or without *H. bilis*) and performed bacterial metatranscriptomic sequencing. Based on principal component analysis of high variance GO terms, we found that the overall microbial transcriptional profile in WT/HET compared to *Was*^*−/−*^ mice differed, even when both inflamed samples containing *H. bilis* and uninflamed samples without *H. bilis* were included (Fig. 5A). This suggests that WASP deficiency is associated with changes in microbial gene expression independent of inflammation. Among the highest variance GO terms, we found that pathways upregulated by the microbiota in *Was*^*−/−*^ mice included those involved in nucleic acid synthesis, such as ribonucleoside monophosphate biosynthetic process, ribose phosphate diphosphokinase activity, nucleotide biosynthetic process, and pyrimidine nucleotide biosynthetic process (Fig. 5B).

### Inflammation in *Was*^*−/−*^ mice is associated with transcriptional changes in ASF457 *M. schaedleri* and ASF519 *P. goldsteinii* that can perpetuate inflammation

We next examined gene expression within each bacterial species individually. To tease apart contributions due to genotype or presence of *H. bilis* versus inflammation, we sequenced samples from an early timepoint (1.5 weeks after *H. bilis* gavage, when inflammation was minimal based on LCN2) and a late timepoint (15 weeks after *H. bilis* gavage, when significant inflammation had been present for weeks). Unexpectedly, despite *H. bilis* playing a critical role in inflammation in *Was*^*−/−*^ mice, no differences were identified in *H. bilis* gene expression in WT compared to *Was*^*−/−*^ mice either at the early or late timepoint (Figs. 5C, S5A). In contrast, ASF457 *M. schaedleri* showed multiple differentially expressed genes in colitic *Was*^*−/−*^ mice harboring ASF and *H. bilis* as compared to non-colitic WT mice harboring the same microbiota (Fig. 5D). These differences were not seen when comparing ASF457 *M. schaedleri* gene expression in the same mice prior to the onset of inflammation (except for *clpB*) (Fig. S5B) or in *Was*^*−/−*^ versus WT mice colonized with ASF alone (Fig. S5D). These data suggest that the transcriptional differences were a result of the inflammatory environment rather than the host genotype or the presence of the pathobiont *H. bilis*. As discussed below, the genes that were upregulated by ASF457 *M. schaedleri* in inflamed *Was*^*−/−*^ mice, such as flagellin, ClpB, DnaK, and molybdopterin-dependent oxidoreductase, are expected to both improve its fitness in stressful environments, such as the inflamed intestine, as well as increase its immunogenicity (Fig. 5D). This supports a bi-directional model in which the inflamed environment induces the expression of genes that allow ASF457 to thrive, and ASF457 upregulates the expression of genes that stimulate the immune system, further perpetuating inflammation.

For ASF519 *P. goldsteinii*, we also observed differences in gene expression in colitic *Was*^*−/−*^ mice harboring ASF and *H. bilis* as compared to non-colitic WT mice harboring the same microbiota (Fig. 5E). As with ASF457 *M. schaedleri*, these differences were not detected when comparing the same mice prior to the onset of inflammation or *Was*^*−/−*^ versus WT mice colonized with ASF alone, suggesting that these differences were due to the inflammatory environment (Fig. S5C,E). However, in contrast to what we observed in ASF457 *M. schaedleri*, ASF519 *P. goldsteinii* in colitic *Was*^*−/−*^ mice had lower expression of genes that would be expected to help it adapt to stressful conditions, such as superoxide dismutase and DNA starvation/stationary phase protection protein (Fig. 5E)*.* This suggests that ASF519 *P. goldsteinii* may have reduced fitness in inflamed colons. Most of the other ASF members showed less-striking differences in gene expression (Fig. S5F-J).

## Discussion

Our study addresses a major area of interest within the field of IBD, which is the cause-and-effect relationship between dysbiosis and intestinal inflammation. We employed a simplified and defined microbiota within a genetically susceptible WASP-deficient host. Some of the strengths of this model include the fact that it requires more than one bacterial species, and the combination of bacteria that induces disease in *Was*^*−/−*^ mice does not do so in WT mice, reflecting some of the complexity seen in human IBD. By using a defined microbiota in a gnotobiotic facility, we were able to minimize differences between experimental groups at the start of the study and monitor for unintentional introduction of new microbes to all or a subset of mice during the study. The limited number of bacteria also allowed us to methodically assess all members of the microbiota throughout the study. Furthermore, this murine model is relevant to human disease, as WASP deficiency in humans results in intestinal inflammation in a subset of patients [22].

*H. bilis* is critical for the development of intestinal inflammation in *Was*^*−/−*^ mice, but it is not pathogenic in and of itself, since mono-colonization of *Was*^*−/−*^ mice does not result in disease. *H. bilis* induced mild inflammation as measured by LCN2, a biomarker of inflammation, within a week after introduction into *Was*^*−/−*^ mice colonized with ASF. The inflammation was temporarily controlled by week 4 but then re-developed by week 6 and persisted thereafter. In contrast, even at early timepoints, WT mice exhibited no significant inflammation after colonization with *H. bilis*. *H. bilis* colonized at similar abundances in WT and *Was*^*−/−*^ mice until week 5, when it reached a significantly higher relative abundance in *Was*^*−/−*^ compared to WT mice. Given that the *Was*^*−/−*^ mice were not inflamed at this timepoint and developed chronic inflammation afterwards, these observations suggest that impaired control of *H. bilis* infection may contribute to the initiation of chronic intestinal inflammation in *Was*^*−/−*^ mice. Our metatranscriptomic analysis indicates that this impaired control is not due to increased production of virulence factors or immunogenic proteins by *H. bilis* in *Was*^*−/−*^ mice.

Given that mucosal-associated bacteria come in closer proximity to the host than luminal bacteria, especially during colitis, it is likely that they are more strongly influenced by the host immune system and contribute more to intestinal inflammation. Consistent with this, a study of treatment-naïve pediatric Crohn’s patients found that microbial dysbiosis was more strongly reflected in the mucosal-associated microbial community as compared to in the stool microbiota [45]. Examination of the mucosal-associated microbiota in our study was limited to the time of sacrifice due to the technical difficulty of obtaining mucosal samples from the proximal colon. In *Was*^*−/−*^ mice colonized with ASF and *H. bilis*, we found that the major members of the mucosal-associated microbiota are *H. bilis*, ASF457 *M. schaedleri*, and ASF519 *P. goldsteinii*. Of these, *H. bilis* relative abundance was not correlated with LCN2 while ASF457 *M. schaedleri* relative abundance was positively correlated with LCN2 and ASF519 *P. goldsteinii* relative abundance trended towards a negative correlation with LCN2. There are several possible explanations for the lack of correlation between *H. bilis* mucosal relative abundance and LCN2. It is possible that *H. bilis* is required to trigger inflammation but is less important for maintaining inflammation. There is precedent for a transient requirement for a pathobiont, as transient colonization of TLR5-deficient mice by adherent-invasive *Escherichia coli* (AIEC) causes chronic inflammation that persists well beyond clearance of the AIEC [46]. In future studies, it would be interesting to develop a method to selectively deplete *H. bilis* after the onset of inflammation in order to determine whether it is still required in later stages of disease. Another possibility is that *H. bilis* only needs to be present above a certain threshold abundance in the mucus to support inflammation and more *H. bilis* does not result in more inflammation. Yet another possibility is that the absolute abundance of *H. bilis* does in fact correlate with inflammation but because we are measuring relative abundance, this increase is masked by changes in the abundance of other bacteria such as ASF457 *M. schaedleri*.

Our study indicates that ASF457 *M. schaedleri* may act as a pathobiont in *Was*^*−/−*^ mice once inflammation has been initiated by another trigger, such as *H. bilis*, and it has been shown to act as a pathobiont in another model of intestinal inflammation [29]. The positive correlation between mucosal ASF457 *M. schaedleri* relative abundance and LCN2 may reflect a bi-directional relationship in which inflammation leads to expansion of this population due to upregulation of genes that confer improved fitness, and expansion of this population further perpetuates inflammation due to upregulation of genes that stimulate the immune system. The identities of the genes that were upregulated in ASF457 *M. schaedleri* in colitic *Was*^*−/−*^ mice after but not prior to the onset of inflammation support this hypothesis. For example, flagellin levels have been reported to be elevated in several murine models of intestinal inflammation, and flagellin can activate pro-inflammatory gene expression via TLR5 and the NLRC4 inflammasome [46–49]. Flagellin is also a dominant target of the immune system in Crohn’s disease [50]. ClpB and DnaK are chaperones that work together to solubilize and refold protein aggregates that can form during stress [51, 52]. They have also been shown to be important for motility and virulence in several different bacteria, and DnaK is immunogenic [53–58]. Furthermore, levels of DnaK and ClpB are increased in the intestinal bacterial proteomes of Crohn’s patients [59]. Molybdenum-cofactor-dependent metabolic pathways have been shown to be upregulated in inflammation-associated microbiota, and mutation of a gene important for the biosynthesis of molybdenum cofactor results in impaired fitness of several bacterial species under inflammatory conditions [60]. Notably, inhibition of molybdenum cofactor activity led to the abolishment of the expansion of *Enterobacteriaceae* and *Deferribacteraceae* (of which ASF457 *M. schaedleri* is a member) and amelioration of disease in dextran sulfate sodium-exposed mice [61]. Thus, our study suggests that in inflammatory environments, ASF457 *M. schaedleri* upregulates genes that are predicted to increase its fitness, virulence, and immunogenicity, allowing it to increase in abundance and induce continued inflammation. In future studies, it will be interesting to test whether co-colonization of *Was*^*−/−*^ mice with *H. bilis* and ASF457 *M. schaedleri* is sufficient to cause intestinal inflammation.

ASF519 *P. goldsteinii* relative abundance was negatively correlated with LCN2, suggesting a possible protective role for this organism. Indeed, oral treatment of obese mice on a high-fat diet with *P. goldsteinii* led to decreased expression of IL-1β and increased expression of IL-10 in the colon as well as reduced intestinal permeability [39]. One mechanism for mediating these beneficial effects may be through the production of short-chain fatty acids [30]. Our metatranscriptomic analysis revealed that ASF519 *P. goldsteinii* expressed higher levels of superoxide dismutase and DNA starvation/stationary phase protection protein, which are important for adaptation to various types of stress, in WT mice compared to colitic *Was*^*−/−*^ mice [62–64]. Thus, this bacterial species may have a relative fitness disadvantage in inflamed colons. Taken together, these data suggest that ASF519 *P. goldsteinii* abundance may decrease in inflammatory conditions due to relatively low expression of genes important for stress response, and the loss of this protective species may in turn worsen colitis.

## Conclusions

In summary, our data support a model in which *H. bilis* serves as an initial trigger for inflammation in WASP-deficient mice, and this inflammatory environment supports the expansion of a second pathobiont, ASF457 *M. schaedleri*, which perpetuates inflammation. At the same time, ASF519 *P. goldsteinii* is not well-adapted for survival in the inflamed colon, and the loss of this protective species further exacerbates colitis. Our study highlights several key concepts that should be taken into consideration when designing future studies. First, the answer to the question of whether dysbiosis causes inflammation or inflammation causes dysbiosis is likely that either may be true depending on the timepoint. This emphasizes the importance of longitudinal studies that begin before the onset of inflammation. Second, microbes can have context-dependent effects, as illustrated by *H. bilis*’s differing effects on T_H_17 cells, ILC3s, and LCN2 depending on host genotype and inflammatory state. Thus, labeling a bacterial taxon broadly as “good” or “pathogenic” without studying its effects in multiple settings may be misleading. Finally, bacteria adapt to their environment by changing their gene expression, which affects how they interact with their host and with other microbes. While inferring functional capacity based on 16S rRNA sequencing or measuring functional potential based on metagenomic sequencing begins to address bacterial function, metatranscriptomic, metabolomic, and proteomic analyses are critical for assessing actual function and behavior in a given setting.

These concepts also have important implications for the design of microbial-based therapeutics. When developing the optimal consortium of bacteria to administer to patients, it will be important to consider how these bacteria will adapt to the inflamed environment they will be delivered into. Indeed, there is growing evidence supporting the importance of recipient characteristics, including inflammatory state, on fecal microbiota transplantation success [10]. Optimizing the intestinal environment to support the growth of beneficial bacteria and induce the desired transcriptional program within these bacteria may be critical to improving the efficacy of microbial-based therapies. Alternatively, beneficial bacteria could be genetically engineered to better survive in the inflamed colon and constitutively express genes that promote tissue repair and amelioration of inflammation.

## Methods and Materials

### Mice

*Was*^*−/−*^ mice were generated as previously described [19]. WT and *Was*^*−/−*^ mice (both on the 129 SvEv background) were maintained under specific pathogen-free (SPF), germ-free, or gnotobiotic conditions at Boston Children’s Hospital (BCH) as indicated. Mice were fed Prolab Isopro RMH 3000. Germ-free status was monitored by both 16S sequencing and culturing on a monthly basis. WT and *Was*^*−/−*^ mice re-derived as *Helicobacter*-free were housed in *Helicobacter-*free conditions at Massachusetts General Hospital (MGH). Mice were between 3-6 months of age at the beginning of the gnotobiotic experiments. WT and *Was*^*−/−*^ experimental groups were balanced by age and sex. For mice receiving *H. bilis*, the *H. bilis* Missouri strain was grown on tryptic soy agar plates with 5% sheep blood (Remel Laboratories, Lenexus, KS) and incubated under microaerobic conditions at 37 °C with a gas mixture of N_2_, Co_2_, and H_2_ (80:10:10). *H. bilis* was grown to an OD600 of 2, and 200 μL were delivered by orogastric gavage into each mouse twice, 2 days apart. Fecal samples were collected longitudinally to monitor for the onset of inflammation and changes in microbial composition. At 20 weeks following *H. bilis* gavage, mice were euthanized, and histology, spleen weights, colonic lamina propria immune cell populations, and mucosal microbiota were assessed. *H. bilis* colonization was confirmed via PCR analysis of fecal pellets. All experiments were conducted after approval from the BCH Institutional Animal Care and Use Committee and the subcommittee on Research Animal Care at Massachusetts General Hospital (MGH).

### Isolation of cells from colonic lamina propria

Colons were harvested and placed in ice-cold phosphate-buffered saline (PBS). Fecal content was removed from the colon, and the colon was opened longitudinally, then cut into approximately 1 cm sections. The colon pieces were incubated in Hank’s balanced salt solution (HBSS, without Ca^2+^ and Mg^2+^) containing 0.5% fetal bovine serum (FBS), 10 mM EDTA, 10 mM HEPES, and 1mM DTT at 37°C with shaking for 20 min twice to remove the epithelial cell layer. After removal of the epithelial layer, tissues were washed in PBS, minced, and digested in HBSS buffer (with Ca^2+^ and Mg^2+^) containing 20% FBS, 1.5 mM CaCl_2_, and collagenase VIII (200 unit/ml) for 45 min at 37°C. The cells were then filtered and washed once in cold PBS.

### Flow cytometry

For T cells, cells isolated from the colonic lamina propria were stimulated with PMA (100 ng/mL) and ionomycin (500 ng/mL) for 4–6 h. Dead cells were excluded using Fixable Viability Dye eFluor 506 (eBioscience). Surface staining was performed using antibodies to CD45 (30-F11), CD3 (145-2C11), and CD4 (GK1.5). Cells were fixed and permeabilized using the Cytofix/Cytoperm Fixation/Permeabilization Solution Kit (BD Biosciences), and intracellular cytokine staining was performed for IL-17A (TC11-18H10.1).

For ILC3s, cells isolated from the colonic lamina propria were rested in RPMI with 10% FBS and penicillin/streptomycin for 1–2 h. IL-7 and IL-23 (both from BioLegend, final concentration 20 ng/mL) were added, and cells were cultured overnight. They were then stimulated with PMA (100 ng/mL) and ionomycin (500 ng/mL) for 4–6 h. Dead cells were excluded using Fixable Viability Dye eFluor 506 (eBioscience). Surface staining was performed using antibodies to CD45 (30-F11), CD19 (1D3/CD19), CD3 (145-2C11), CD11b (M1/70), CD11c (N418), NK1.1 (PK136), Ly6C/G (RB6-8C5), Thy1.2 (30H12), CD127 (A7R34). Cells were fixed and permeabilized using the Cytofix/Cytoperm Fixation/Permeabilization Solution Kit (BD Biosciences), and intracellular cytokine staining was performed for IL-22 (Poly5164).

All antibodies were purchased from BioLegend. Samples were run on a BD LSRFortessa Cell Analyzer and analyzed using Flowjo software, version 10.

### Histology

Tissue from the indicated areas of the colon were harvested and flushed with PBS. Tissue was fixed in 4% paraformaldehyde and embedded in paraffin. Sections were stained for H&E, and images were graded for severity of inflammation using a score that incorporates the following 4 parameters, each graded on a 4-point scale (0 = absent, 1 = mild, 2 = moderate, 3 = severe): mononuclear inflammation, crypt hyperplasia, epithelial injury, and neutrophilic inflammation/crypt abscesses.

### Gut bacteria and mucus staining

Colon sections containing fecal material were fixed in Carnoy’s buffer to preserve the mucus layer and then were paraffin-embedded. 5-μm-thick sections were cut for staining. Bacteria were stained by fluorescence in situ hybridization using a Cy3-conjugated pan-specific EUB338 probe (5′- GCT GCC TCC CGT AGG AGT -3′), which covers more than 90% of the gut bacteria. The sections were counterstained with 4′,6-diamidino-2-phenylindole (DAPI), indicating the colonic epithelium and the lectin Ulex europaeus agglutinin I (*UEA*-*1*), indicating the mucus layer. Cells were imaged on a Zeiss LSM880 confocal Microscope. Original magnification 200× or 630×.

### Fecal lipocalin-2 ELISA

Stool samples were collected by holding the mice and allowing them to defecate directly into sterile tubes. Stool was stored at – 80 °C. One to two stool pellets were weighed and placed in a fresh sterile tube, and 1 mL of PBS + 0.1% Tween20 was added. Tubes were clipped to a horizontal microtube attachment on a Vortex Genie 2 and vortexed at high speed for 5 min. 750 μL of stool homogenate were transferred to a fresh tube and vortexed for an additional 15 min. The samples were then centrifuged at 12,000 rpm for 10 min at 4 °C. The supernatant was transferred to a fresh tube and stored at − 20 °C. LCN2 was measured in the supernatant using the mouse lipocalin-2/NGAL DuoSet ELISA kit (R&D Systems) and normalized to stool weight.

### Fecal DNA extraction and 16S library preparation

Fecal DNA was extracted using the DNeasy PowerSoil Kit for SPF samples. Due to the large number of samples in the ASF experiment, the similar but high throughput DNeasy PowerSoil HTP 96 Kit (384) was used. DNA from samples within the same study that were directly compared were extracted using the same kit. For mucosal samples, approximately 1-cm pieces of the proximal colon were stored in TRIzol Reagent at − 80 °C. After samples were thawed, they were rinsed in PBS and added to the bead tube of the DNeasy PowerSoil Kit (Qiagen). Sixty microliters of Solution C1 (Qiagen DNeasy PowerSoil Kit) were added to each tube, and the tubes were incubated at 70 °C for 10 min. Samples underwent bead beating in a Qiagen TissueLyser II at setting 30 for 2 min. Twenty microliters of proteinase K (Sigma) were added to each tube, and tubes were incubated at 65 °C for 30 min. Samples were then processed as per the DNeasy PowerSoil Kit (Qiagen) protocol beginning after the vortexing step.

The 16S rRNA V4 region was PCR-amplified using primers adapted from the 515F and 806R primers used by the Earth Microbiome Project and modified to include Illumina paired-end adaptors (forward: CTT TCC CTA CAC GAC GCT CTT CCG ATC TGT GCC AGC MGC CGC GGT AA; reverse GGA GTT CAG ACG TGT GCT CTT CCG ATC TGG ACT ACH VGG GTW TCT AAT) [65]. Nextera XT indices (Illumina) were attached to the 16S V4 amplicons during a second PCR step. Both PCR steps were performed using 5PRIME HotMasterMix (Quantabio). Cycling conditions for the first step were: 94°C for 3 min; 20 cycles of 94 °C for 45 s (28 cycles for mucosal samples), 50 °C for 60 s, and 72 °C for 90 s; then 72 °C for 10 min. For mucosal samples, two separate 16S rRNA PCR reactions were performed for each sample, and the products from the two independent first-round PCR reactions were combined as input for the second PCR round to attach indices. Cycling conditions for the second step were 94 °C for 3 min; 5 cycles of 94°C for 45 s, 65 °C for 60 s, and 72°C for 90 s; then 72 °C for 10 min. Amplicons were purified and normalized using the SequalPrep Normalization Plate Kit (Invitrogen). Paired-end sequencing was done on the Miseq platform using the 300-cycle V2 kit for stool samples and the 300-cycle Micro kit for mucosal samples. Sequencing of SPF fecal samples yielded a mean of 313,000 (standard deviation 95,000) read pairs per sample. Sequencing of ASF fecal samples yielded a mean of 18,000 (standard deviation 4,000) read pairs per sample. Sequencing of mucosal samples yielded a mean of 72,000 (standard deviation 15,000) read pairs per sample.

### Analysis of 16S sequencing

The R package, dada2 (version 1.14.1), was used to process 16S amplicon sequence data to generate an amplicon sequence variant (ASV) table [66]. Specifically, reads were filtered and trimmed using the filterAndTrim command with options trimLeft=c(19,20), truncLen=c(150,150), maxN=0, maxEE=c(2,2), truncQ=2, rm.phix=TRUE. Sequence error models for forward and reverse reads were learned from the filtered, trimmed reads using the learnErrors command. Sequence errors were then corrected using the dada function, producing denoised reads that were then merged using the mergePairs function with options minOverlap=7, maxMismatch=1. For the mucosal bacteria dataset, reads were slightly shorter and therefore minOverlap=4 was used. An ASV table was then generated using the makeSequenceTable function. Chimeras were detected and removed using the removeBimeraDenovo function using the “consensus” method.

For samples from SPF mice, taxonomic assignment was performed using the dada2 functions assignTaxonomy and addSpecies using version 16 of the Ribosomal Database Project training set [67]. For samples from mice colonized with ASF with or without *H. bilis*, ASVs were identified by comparison to the corresponding 16S sequences found in reference genomes using the search_pcr command of USEARCH version 10 [68]. The NCBI accession numbers of the reference genomes used are as follows: NZ_CP019645.1 (*Helicobacter bilis* strain AAQJH); NZ_KB822565.1 (*Clostridium* sp. ASF356); NZ_KB822413.1 (*Lactobacillus* sp. ASF360); NZ_KB822402.1 (*Lactobacillus murinus* ASF361); NZ_KI530573.1 (*Mucispirillum schaedleri* ASF457); NZ_KB822471.1 (*Eubacterium plexicaudatum* ASF492); NZ_KI535318.1 (*Firmicutes* bacterium ASF500); NZ_RHJS01000002.1 (*Schaedlerella arabinosiphila* ASF502); NZ_KB822571.1 (*Parabacteroides* sp. ASF519).

The R package phyloseq version 1.30.0 was used for further analysis [69]. All beta diversity/principal coordinates analyses were performed after rarefaction to even sequencing depth. The rarefaction depth used was the minimum read depth among the samples involved in the analysis, unless this was less than 10,000, in which case 10,000 reads per sample were used.

### Fecal RNA extraction and library preparation

For each mouse, a single fecal pellet was deposited into a Qiagen PowerBead glass 0.1 mm tube. 1 mL of Invitrogen TRIzol Reagent was added to each tube. The tubes were sealed and clipped to a horizontal microtube attachment on a Vortex Genie 2 and vortexed at high speed for 20 min. The tubes were centrifuged quickly, and 200 μL of chloroform was added. The tubes were then vortexed for 1 minute, allowed to homogenate at room temperature for 3 min, and then centrifuged at 4 °C for 15 min. The upper aqueous layer was carefully transferred to a 1.5-mL Eppendorf tube. A 1:1 ratio of 70% ethanol was added to each tube. The tubes were then sealed and vortexed for 1 minute followed by centrifugation at room temperature for 1 minute. The samples were then passed through Qiagen RNeasy Mini Kit spin columns, 600 μL at a time, and the flow-through was discarded. RNA was extracted as per the Qiagen RNeasy Mini Kit protocol, including the on-column DNase digestion step. 80% ethanol was used in place of RPE for the second RPE wash step. RNA was eluted by adding 50 μL of RNAse-free water to the center of each spin column membrane and incubating at room temperature for 5 min. The tubes were centrifuged at maximum speed at room temperature for 30 s. The captured eluate was then transferred to the center of each spin column membrane, and the spin columns were centrifuged at maximum speed for 2 min. The extracted RNA was quantified using the Invitrogen Qubit RNA HS Assay Kit, and the quality of the RNA was assessed using an Agilent RNA 6000 Pico Kit.

Fifty microliters of Beckman Coulter RNAClean XP beads were added to the 50 μl of extracted RNA for each sample. Samples were washed per Beckman Coulter’s wash protocol and eluted in 20 μl. Bacterial ribosomal RNA was removed using the Invitrogen RiboMinus Transcriptome Isolation Bacteria Kit, and the ribo-depleted RNA was eluted in 10 μl. Sequencing libraries were generated using the Illumina TruSeq Stranded Total RNA Library Prep Gold kit to ensure host ribosomal RNA depletion and mitochondrial RNA depletion. TruSeq RNA indexes were used to barcode samples.

cDNA libraries were washed using Beckman Coulter AMPure XP magnetic beads. Library quality and size verification were performed using the PerkinElmer LabChip GXII instrument with the DNA 1K Reagent Kit. Library concentrations were quantified using the Quant-iT dsDNA High Sensitivity Assay Kit using a Promega GloMax plate reader. Library molarity was calculated based on library peak size and concentration. Libraries were normalized to 2 nM using the PerkinElmer Zephyr G3 NGS Workstation and pooled together using the same volume across all normalized libraries into a 1.5-ml Eppendorf DNA tube. Pooled libraries were sequenced on an Illumina NextSeq500 high-output 75-bp single-read sequencing run. Between 3.4 million and 26.7 million 75-bp raw reads per sample were generated (median 16.9 million reads).

### Analysis of metatranscriptomic sequencing

Raw sequence reads were deduplicated using the dedup function of samtools [70]. Deduplicated reads served as the input to both gene-level and pathway-level analyses.

For gene-based analyses, deduplicated reads were mapped using Bowtie2 [71] to an index containing the reference genomes of *Helicobacter bilis* and the ASF members (see Analysis of 16S Sequencing for accession numbers). The resulting mapped reads (BAM files) were then split into separate files according to species. Reads mapped per gene were counted with the Rsubread packag e[72], using the annotations (GFF files) provided with the reference assemblies. Reads mapping to ribosomal genes (annotated with gene_biotype = rRNA) were removed. All data associated with a given bacterial species were then grouped together, yielding the equivalent of a bulk RNA dataset for each species. Further differential expression analysis was performed using DESeq2 [73]. Principal components analysis was performed after applying the DESeq2 variance stabilization transformation.

For pathway-based analyses, ribosomal reads were removed from the deduplicated reads using the bbduk tool from the bbtools suite [74] with kmer length 27. First, reads mapping to ribosomal small subunit sequences in the SILVA SSU database version 132 [75] were removed. Reads mapping to large subunit sequences in SILVA LSU database version 132 were then removed. Prior to use, these databases were modified to replace nucleotides U with T. The filtered reads were then analyzed using a beta version of HUMAnN3 [76], yielding pathway abundances normalized as copies per million (CPM). For the purposes of principal coordinates analysis, high-variance pathways (GO terms) were identified as follows. Pathways were filtered to remove those present in 3 or fewer samples. Pathway abundances were log-transformed (using a pseudocount of 0.1 CPM). It was noted that the mean/variance relationship of the resulting transformed values was strongly confounded by pathway prevalence (that is, by the number of samples in which each pathway was detected). This confounding effect was taken into account by grouping pathways according to the number of samples with each pathway detected and performing a linear regression in which the independent variable was the mean log-transformed CPM and the dependent variable was the corresponding standard deviation. The resulting residuals were then *z*-scored in order to normalize across all pathways. Pathways with high *z*-scores were considered to have high variance, taking into account overall expression and pathway prevalence. Principal component analysis (PCA) of GO term expression was performed using the log-transformed CPM values of the 30 highest variance GO terms. Clustering of samples according to genotype was assessed using PERMANOVA as implemented by the adonis function of the vegan R package [77] using Euclidean distance.

### Statistical analyses

Statistical tests appropriate for each type of data are indicated in the figure legends and were performed using R (R Core Team 2021) [78] or GraphPad Prism (version 9.1.2). For box-and-whisker plots, boxes show 25th, 50th, and 75th percentiles, and whiskers include all data within 1.5 box lengths of the box. Outliers beyond the whiskers are shown as points. Correlations between log-transformed LCN2 levels and bacterial relative abundances were assessed using the Pearson correlation test in cases when one sample per mouse was used. When multiple samples per mouse were involved, these correlations were tested by fitting linear mixed-effects models using the lme4 R package [79] with random mouse effects.

## Supplementary Information

**Additional file 1: Fig. S1.** WASP deficiency results in altered composition of the fecal microbiota. Fecal microbial composition of *Was*^*-/-*^ (n=5) and WT (n=3) mice raised under SPF conditions with weekly bedding exchanges was analyzed monthly between 4 and 20 weeks of age by 16S rRNA gene sequencing. **(A)** Microbial relative abundances at the phylum level. **(B)** Relative abundance of the phylum Deferribacteres at each timepoint by genotype. Statistics performed using the DESeq2 R package and adjusted for multiple taxa comparisons. * *p* < 0.05, ** *p* < 0.01, *** *p* < 0.001. **Fig. S2.** Establishing a reductionist model to study the role of the microbiota in the development of intestinal inflammation. **(A)** Experimental design. **(B)** Fecal microbial composition of donor mice harboring the ASF consortium and recipient ex-germ-free mice after 2 months of co-housing (females) or bedding exchanges (males) with donors. Composition assessed based on 16S rRNA sequencing. **(C)** Quantitative histological colitis scores 20 weeks after gavage with *H. bilis* in the cecum, proximal colon, and distal colon*.* **(D)** Gating strategy for IL-17A^+^ CD4 T cells. **(E)** Gating strategy for IL-22^+^ ILC3s. Lin includes CD3, CD19, CD11b, CD11c, NK1.1, Ly6C, Ly6G. **Fig. S3.** Correlations between intestinal inflammation and fecal microbial composition. (**A**-**B**) Germ free WT/HET (n=11) and *Was*^*-/-*^ (n=8) mice colonized with the ASF community but not gavaged with *H. bilis* served as a control group. Fecal LCN2 **(A)** and absence of *H. bilis* **(B)** were monitored serially. **(C)** In mice that received *H. bilis*, *H. bilis* relative abundance is shown based on whether the mouse was in a cage that contained both genotypes (co-housed) or only one genotype (not co-housed). (**D-I**) Correlations between log-transformed fecal LCN2 and relative abundances of the indicated ASF members in mice of the indicated genotype colonized with ASF and *H. bilis* for all timepoints. Tests for linear dependence of log-transformed LCN2 on the relative abundance of each bacterial species was done using a linear mixed effects model, taking into account a mouse-specific random effect. Wilcoxon rank sum test was used for **(A)** and **(C)**. * *p* < 0.05, *** *p* < 0.001. **Fig. S4.** Correlations between intestinal inflammation and mucosal microbial composition. **(A-H)** Germ free WT/HET (n=17) and *Was*^*-/-*^ (n=13) mice were colonized with the ASF community and then gavaged with *H. bilis*. The mucosal-associated microbiota was assessed at 20 weeks post infection. Pearson correlations between log-transformed fecal LCN2 and mucosal relative abundances of the indicated ASF members are shown within mice of the indicated genotypes. **Fig. S5.** Metatranscriptomic analysis of ASF members in inflamed and uninflamed states. Germ-free WT/HET and *Was*^*-/-*^ mice were colonized with the ASF community and a subset were gavaged with *H. bilis*. Feces from 3 mice of each genotype/microbiota combination (WT/HET or *Was*^*-/-*^ with or without *H. bilis*) at 1.5 weeks and 15 weeks after *H. bilis* gavage were subjected to bacterial metatranscriptomic sequencing. (**A-J**) Volcano plots of corrected p-values vs log_2_(FC) for genes expressed by the indicated bacteria at 15 weeks after *H. bilis* gavage, except for **(A-C)**, which are from 1.5 weeks after *H. bilis* infection. Plots compare gene expression of the specified bacteria between host genotypes within the ASF-only group or the ASF + *H. bilis* group as indicated. Dots on the right side of each plot represent genes that are more highly expressed in *Was*^*-/-*^, and dots on the left side indicate genes that are more highly expressed in WT/HET. Red dots represent genes that are significantly differentially expressed as determined by the DESeq2 Wald test.

## Data Availability

The datasets generated during and/or analyzed during the current study are available at the following NCBI BioProject accession numbers: PRJNA729789, PRJNA729739, PRJNA729746, PRJNA729753, PRJNA729783.

## Abbreviations

AIEC: adherent-invasive *Escherichia coli*
ASF: Altered Schaedler flora
ASV: Amplicon sequence variant
CPM: Copies per million
FBS: Fetal bovine serum
HET: Heterozygous
IBD: Inflammatory bowel disease
ILC3: Group 3 innate lymphoid cells
LCN2: Lipocalin-2
PBS: Phosphate-buffered saline
PCA: Principal component analysis
SPF: Specific pathogen-free
T_H_17: T helper 17
T_reg_: Regulatory T cells
UEA-1: *Ulex europaeus* agglutinin I
WASP: Wiskott-Aldrich syndrome protein
WT: Wild-type

## References

[1] Jostins L, Ripke S, Weersma RK, Duerr RH, McGovern DP, Hui KY (2012). Host-microbe interactions have shaped the genetic architecture of inflammatory bowel disease. Nature..

[2] Liu JZ, van Sommeren S, Huang H, Ng SC, Alberts R, Takahashi A (2015). Association analyses identify 38 susceptibility loci for inflammatory bowel disease and highlight shared genetic risk across populations. Nat Genet.

[3] de Lange KM, Moutsianas L, Lee JC, Lamb CA, Luo Y, Kennedy NA, Jostins L, Rice DL, Gutierrez-Achury J, Ji SG, Heap G, Nimmo ER, Edwards C, Henderson P, Mowat C, Sanderson J, Satsangi J, Simmons A, Wilson DC, Tremelling M, Hart A, Mathew CG, Newman WG, Parkes M, Lees CW, Uhlig H, Hawkey C, Prescott NJ, Ahmad T, Mansfield JC, Anderson CA, Barrett JC (2017). Genome-wide association study implicates immune activation of multiple integrin genes in inflammatory bowel disease. Nat Genet.

[4] Pittayanon R, Lau JT, Leontiadis GI, Tse F, Yuan Y, Surette M (2020). Differences in gut microbiota in patients with vs without inflammatory bowel diseases: a systematic review. Gastroenterology.

[5] Khan KJ, Ullman TA, Ford AC, Abreu MT, Abadir A, Marshall JK (2011). Antibiotic therapy in inflammatory bowel disease: a systematic review and meta-analysis. Official J Am Coll Gastroenterol | ACG.

[6] D’Haens GR, Geboes K, Peeters M, Baert F, Penninckx F, Rutgeerts P (1998). Early lesions of recurrent Crohn’s disease caused by infusion of intestinal contents in excluded ileum. Gastroenterology..

[7] Rutgeerts P, Goboes K, Peeters M, Hiele M, Penninckx F, Aerts R (1991). Effect of faecal stream diversion on recurrence of Crohn’s disease in the neoterminal ileum. Lancet..

[8] Paramsothy S, Paramsothy R, Rubin DT, Kamm MA, Kaakoush NO, Mitchell HM, Castaño-Rodríguez N (2017). Faecal microbiota transplantation for inflammatory bowel disease: a systematic review and meta-analysis. J Crohns Colitis.

[9] Imdad A, Nicholson MR, Tanner-Smith EE, Zackular JP, Gomez-Duarte OG, Beaulieu DB (2018). Fecal transplantation for treatment of inflammatory bowel disease. Cochrane Database Syst Rev.

[10] Danne C, Rolhion N, Sokol H (2021). Recipient factors in faecal microbiota transplantation: one stool does not fit all. Nat Rev Gastroenterol Hepatol.

[11] Strober W, Fuss IJ, Blumberg RS (2002). The immunology of mucosal models of inflammation. Annu Rev Immunol.

[12] Rogala AR, Oka A, Sartor RB (2020). Strategies to dissect host-microbial immune interactions that determine mucosal homeostasis vs. intestinal inflammation in gnotobiotic mice. Front Immunol.

[13] Garrett WS, Lord GM, Punit S, Lugo-Villarino G, Mazmanian SK, Ito S, Glickman JN, Glimcher LH (2007). Communicable ulcerative colitis induced by T-bet deficiency in the innate immune system. Cell..

[14] Schaubeck M, Clavel T, Calasan J, Lagkouvardos I, Haange SB, Jehmlich N, Basic M, Dupont A, Hornef M, Bergen M, Bleich A, Haller D (2016). Dysbiotic gut microbiota causes transmissible Crohn’s disease-like ileitis independent of failure in antimicrobial defence. Gut..

[15] Smith PM, Howitt MR, Panikov N, Michaud M, Gallini CA, Bohlooly-Y M, Glickman JN, Garrett WS (2013). The microbial metabolites, short-chain fatty acids, regulate colonic Treg cell homeostasis. Science..

[16] Arpaia N, Campbell C, Fan X, Dikiy S, van der Veeken J, deRoos P (2013). Metabolites produced by commensal bacteria promote peripheral regulatory T-cell generation. Nature.

[17] Furusawa Y, Obata Y, Fukuda S, Endo TA, Nakato G, Takahashi D, Nakanishi Y, Uetake C, Kato K, Kato T, Takahashi M, Fukuda NN, Murakami S, Miyauchi E, Hino S, Atarashi K, Onawa S, Fujimura Y, Lockett T, Clarke JM, Topping DL, Tomita M, Hori S, Ohara O, Morita T, Koseki H, Kikuchi J, Honda K, Hase K, Ohno H (2013). Commensal microbe-derived butyrate induces the differentiation of colonic regulatory T cells. Nature..

[18] Mazmanian SK, Round JL, Kasper DL (2008). A microbial symbiosis factor prevents intestinal inflammatory disease. Nature..

[19] Snapper SB, Rosen FS, Mizoguchi E, Cohen P, Khan W, Liu CH, Hagemann TL, Kwan SP, Ferrini R, Davidson L, Bhan AK, Alt FW (1998). Wiskott-Aldrich syndrome protein-deficient mice reveal a role for WASP in T but not B cell activation. Immunity..

[20] Derry JM, Ochs HD, Francke U (1994). Isolation of a novel gene mutated in Wiskott-Aldrich syndrome. Cell..

[21] Thrasher AJ, Burns SO (2010). WASP: a key immunological multitasker. Nat Rev Immunol.

[22] Dupuis-Girod S, Medioni J, Haddad E, Quartier P, Cavazzana-Calvo M, Le Deist F (2003). Autoimmunity in Wiskott-Aldrich syndrome: risk factors, clinical features, and outcome in a single-center cohort of 55 patients. Pediatrics..

[23] Nguyen DD, Maillard MH, Cotta-de-Almeida V, Mizoguchi E, Klein C, Fuss I (2007). Lymphocyte-dependent and Th2 cytokine-associated colitis in mice deficient in Wiskott-Aldrich syndrome protein. Gastroenterology..

[24] Nguyen DD, Muthupalani S, Goettel JA, Eston MA, Mobley M, Taylor NS, McCabe A, Marin R, Snapper SB, Fox JG (2013). Colitis and colon cancer in WASP-deficient mice require helicobacter species. Inflamm Bowel Dis.

[25] Schaedler RW, Dubs R, Costello R (1965). Association of germfree mice with bacteria isolated from normal mice. J Exp Med.

[26] Dewhirst FE, Chien CC, Paster BJ, Ericson RL, Orcutt RP, Schauer DB, Fox JG (1999). Phylogeny of the defined murine microbiota: altered Schaedler flora. Appl Environ Microbiol.

[27] Wannemuehler MJ, Overstreet A-M, Ward DV, Phillips GJ. Draft genome sequences of the altered Schaedler flora, a defined bacterial community from gnotobiotic mice. Genome Announc. 2014;2(2). 10.1128/genomeA.00287-14. 10.1128/genomeA.00287-14 PMC3983311 24723722

[28] Berry D, Schwab C, Milinovich G, Reichert J, Ben Mahfoudh K, Decker T, Engel M, Hai B, Hainzl E, Heider S, Kenner L, Müller M, Rauch I, Strobl B, Wagner M, Schleper C, Urich T, Loy A (2012). Phylotype-level 16S rRNA analysis reveals new bacterial indicators of health state in acute murine colitis. ISME J.

[29] Caruso R, Mathes T, Martens EC, Kamada N, Nusrat A, Inohara N, et al. A specific gene-microbe interaction drives the development of Crohn’s disease-like colitis in mice. Sci Immunol. 2019;4(34). 10.1126/sciimmunol.aaw4341. 10.1126/sciimmunol.aaw4341 PMC8882361 31004013

[30] Biggs MB, Medlock GL, Moutinho TJ, Lees HJ, Swann JR, Kolling GL, Papin JA (2017). Systems-level metabolism of the altered Schaedler flora, a complete gut microbiota. ISME J.

[31] Wymore Brand M, Wannemuehler MJ, Phillips GJ, Proctor A, Overstreet A-M, Jergens AE, Orcutt RP, Fox JG (2015). The altered Schaedler flora: continued applications of a defined murine microbial community. ILAR J.

[32] Chai JN, Peng Y, Rengarajan S, Solomon BD, Ai TL, Shen Z, et al. Helicobacter species are potent drivers of colonic T cell responses in homeostasis and inflammation. Sci Immunol. 2017;2(13). 10.1126/sciimmunol.aal5068. 10.1126/sciimmunol.aal5068 PMC5684094 28733471

[33] Xu M, Pokrovskii M, Ding Y, Yi R, Au C, Harrison OJ, Galan C, Belkaid Y, Bonneau R, Littman DR (2018). c-MAF-dependent regulatory T cells mediate immunological tolerance to a gut pathobiont. Nature..

[34] Danne C, Ryzhakov G, Martínez-López M, Ilott NE, Franchini F, Cuskin F (2017). A Large polysaccharide produced by Helicobacter hepaticus induces an anti-inflammatory gene signature in macrophages. Cell Host Microbe.

[35] Sugimoto K, Ogawa A, Mizoguchi E, Shimomura Y, Andoh A, Bhan AK, Blumberg RS, Xavier RJ, Mizoguchi A (2008). IL-22 ameliorates intestinal inflammation in a mouse model of ulcerative colitis. J Clin Invest.

[36] Sawa S, Lochner M, Satoh-Takayama N, Dulauroy S, Bérard M, Kleinschek M, Cua D, di Santo JP, Eberl G (2011). RORγt+ innate lymphoid cells regulate intestinal homeostasis by integrating negative signals from the symbiotic microbiota. Nat Immunol.

[37] Mielke LA, Jones SA, Raverdeau M, Higgs R, Stefanska A, Groom JR, Misiak A, Dungan LS, Sutton CE, Streubel G, Bracken AP, Mills KHG (2013). Retinoic acid expression associates with enhanced IL-22 production by γδ T cells and innate lymphoid cells and attenuation of intestinal inflammation. J Exp Med.

[38] Bostick JW, Wang Y, Shen Z, Ge Y, Brown J, Chen Z-ME, Mohamadzadeh M, Fox JG, Zhou L (2019). Dichotomous regulation of group 3 innate lymphoid cells by nongastric Helicobacter species. Proc Natl Acad Sci U S A.

[39] Wu T-R, Lin C-S, Chang C-J, Lin T-L, Martel J, Ko Y-F, Ojcius DM, Lu CC, Young JD, Lai HC (2019). Gut commensal Parabacteroides goldsteinii plays a predominant role in the anti-obesity effects of polysaccharides isolated from Hirsutella sinensis. Gut..

[40] Li H, Limenitakis JP, Fuhrer T, Geuking MB, Lawson MA, Wyss M, Brugiroux S, Keller I, Macpherson JA, Rupp S, Stolp B, Stein JV, Stecher B, Sauer U, McCoy KD, Macpherson AJ (2015). The outer mucus layer hosts a distinct intestinal microbial niche. Nat Commun.

[41] Zoetendal EG, von Wright A, Vilpponen-Salmela T, Ben-Amor K, Akkermans ADL, de Vos WM (2002). Mucosa-associated bacteria in the human gastrointestinal tract are uniformly distributed along the colon and differ from the community recovered from feces. Appl Environ Microbiol.

[42] Johansson MEV, Gustafsson JK, Holmén-Larsson J, Jabbar KS, Xia L, Xu H, Ghishan FK, Carvalho FA, Gewirtz AT, Sjövall H, Hansson GC (2014). Bacteria penetrate the normally impenetrable inner colon mucus layer in both murine colitis models and patients with ulcerative colitis. Gut..

[43] Robertson BR, O’Rourke JL, Neilan BA, Vandamme P, On SLW, Fox JG (2005). Mucispirillum schaedleri gen. nov., sp. nov., a spiral-shaped bacterium colonizing the mucus layer of the gastrointestinal tract of laboratory rodents. Int J Syst Evol Microbiol.

[44] Fox JG (2002). The non-H pylori helicobacters: their expanding role in gastrointestinal and systemic diseases. Gut..

[45] Gevers D, Kugathasan S, Denson LA, Vázquez-Baeza Y, Van Treuren W, Ren B (2014). The treatment-naive microbiome in new-onset Crohn’s disease. Cell Host Microbe.

[46] Chassaing B, Koren O, Carvalho FA, Ley RE, Gewirtz AT (2014). AIEC pathobiont instigates chronic colitis in susceptible hosts by altering microbiota composition. Gut..

[47] Chassaing B, Koren O, Goodrich JK, Poole AC, Srinivasan S, Ley RE, Gewirtz AT (2015). Dietary emulsifiers impact the mouse gut microbiota promoting colitis and metabolic syndrome. Nature..

[48] Cullender TC, Chassaing B, Janzon A, Kumar K, Muller CE, Werner JJ, Angenent LT, Bell ME, Hay AG, Peterson DA, Walter J, Vijay-Kumar M, Gewirtz AT, Ley RE (2013). Innate and adaptive immunity interact to quench microbiome flagellar motility in the gut. Cell Host Microbe.

[49] Zhao Y, Yang J, Shi J, Gong Y-N, Lu Q, Xu H, Liu L, Shao F (2011). The NLRC4 inflammasome receptors for bacterial flagellin and type III secretion apparatus. Nature..

[50] Lodes MJ, Cong Y, Elson CO, Mohamath R, Landers CJ, Targan SR, Fort M, Hershberg RM (2004). Bacterial flagellin is a dominant antigen in Crohn disease. J Clin Invest.

[51] Zolkiewski M (1999). ClpB cooperates with DnaK, DnaJ, and GrpE in suppressing protein aggregation. A novel multi-chaperone system from Escherichia coli. J Biol Chem.

[52] Goloubinoff P, Mogk A, Zvi AP, Tomoyasu T, Bukau B (1999). Sequential mechanism of solubilization and refolding of stable protein aggregates by a bichaperone network. Proc Natl Acad Sci U S A.

[53] Jain S, Smyth D, O’Hagan BMG, Heap JT, McMullan G, Minton NP, Ternan NG (2017). Inactivation of the dnaK gene in Clostridium difficile 630 Δerm yields a temperature-sensitive phenotype and increases biofilm-forming ability. Sci Rep.

[54] Takaya A, Tomoyasu T, Matsui H, Yamamoto T (2004). The DnaK/DnaJ chaperone machinery of Salmonella enterica serovar Typhimurium is essential for invasion of epithelial cells and survival within macrophages, leading to systemic infection. Infect Immun.

[55] Alam A, Golovliov I, Javed E, Kumar R, Ådén J, Sjöstedt A (2020). Dissociation between the critical role of ClpB of Francisella tularensis for the heat shock response and the DnaK interaction and its important role for efficient type VI secretion and bacterial virulence. PLoS Pathog.

[56] Lourdault K, Cerqueira GM, Wunder EA, Picardeau M (2011). Inactivation of clpB in the pathogen Leptospira interrogans reduces virulence and resistance to stress conditions. Infect Immun.

[57] Shi W, Zhou Y, Wild J, Adler J, Gross CA (1992). DnaK, DnaJ, and GrpE are required for flagellum synthesis in Escherichia coli. J Bacteriol.

[58] Fourie KR, Wilson HL (2020). Understanding GroEL and DnaK stress response proteins as antigens for bacterial diseases. Vaccines (Basel).

[59] Juste C, Kreil DP, Beauvallet C, Guillot A, Vaca S, Carapito C, Mondot S, Sykacek P, Sokol H, Blon F, Lepercq P, Levenez F, Valot B, Carré W, Loux V, Pons N, David O, Schaeffer B, Lepage P, Martin P, Monnet V, Seksik P, Beaugerie L, Ehrlich SD, Gibrat JF, van Dorsselaer A, Doré J (2014). Bacterial protein signals are associated with Crohn’s disease. Gut..

[60] Hughes ER, Winter MG, Duerkop BA, Spiga L, Furtado de Carvalho T, Zhu W (2017). Microbial respiration and formate oxidation as metabolic signatures of inflammation-associated dysbiosis. Cell Host Microbe.

[61] Zhu W, Winter MG, Byndloss MX, Spiga L, Duerkop BA, Hughes ER, Büttner L, de Lima Romão E, Behrendt CL, Lopez CA, Sifuentes-Dominguez L, Huff-Hardy K, Wilson RP, Gillis CC, Tükel Ç, Koh AY, Burstein E, Hooper LV, Bäumler AJ, Winter SE (2018). Precision editing of the gut microbiota ameliorates colitis. Nature..

[62] Broxton CN, Culotta VC (2016). SOD Enzymes and microbial pathogens: surviving the oxidative storm of infection. PLoS Pathog.

[63] Almirón M, Link AJ, Furlong D, Kolter R (1992). A novel DNA-binding protein with regulatory and protective roles in starved Escherichia coli. Genes Dev.

[64] Karas VO, Westerlaken I, Meyer AS (2015). The DNA-binding protein from starved cells (Dps) utilizes dual functions to defend cells against multiple stresses. J Bacteriol.

[65] Scheiman J, Luber JM, Chavkin TA, MacDonald T, Tung A, Pham L-D, Wibowo MC, Wurth RC, Punthambaker S, Tierney BT, Yang Z, Hattab MW, Avila-Pacheco J, Clish CB, Lessard S, Church GM, Kostic AD (2019). Meta-omics analysis of elite athletes identifies a performance-enhancing microbe that functions via lactate metabolism. Nat Med.

[66] Callahan BJ, McMurdie PJ, Rosen MJ, Han AW, Johnson AJA, Holmes SP (2016). DADA2: High-resolution sample inference from Illumina amplicon data. Nat Methods.

[67] Cole JR, Wang Q, Fish JA, Chai B, McGarrell DM, Sun Y (2014). Ribosomal Database Project: data and tools for high throughput rRNA analysis. Nucleic Acids Res.

[68] Edgar RC (2010). Search and clustering orders of magnitude faster than BLAST. Bioinformatics..

[69] McMurdie PJ, Holmes S (2013). phyloseq: an R package for reproducible interactive analysis and graphics of microbiome census data. PLoS ONE.

[70] Li H, Handsaker B, Wysoker A, Fennell T, Ruan J, Homer N, Marth G, Abecasis G, Durbin R, 1000 Genome Project Data Processing Subgroup (2009). The Sequence Alignment/Map format and SAMtools. Bioinformatics..

[71] Langmead B, Salzberg SL (2012). Fast gapped-read alignment with Bowtie 2. Nat Methods.

[72] Liao Y, Smyth GK, Shi W (2019). The R package Rsubread is easier, faster, cheaper and better for alignment and quantification of RNA sequencing reads. Nucleic Acids Res.

[73] Love MI, Huber W, Anders S (2014). Moderated estimation of fold change and dispersion for RNA-seq data with DESeq2. Genome Biol.

[74] BBTools [Internet]. Available from: https://jgi.doe.gov/data-and-tools/bbtools/. Accessed 6 May 2021.

[75] Quast C, Pruesse E, Yilmaz P, Gerken J, Schweer T, Yarza P, Peplies J, Glöckner FO (2013). The SILVA ribosomal RNA gene database project: improved data processing and web-based tools. Nucleic Acids Res.

[76] Franzosa EA, McIver LJ, Rahnavard G, Thompson LR, Schirmer M, Weingart G (2018). Species-level functional profiling of metagenomes and metatranscriptomes. Nat Methods.

[77] Oksanen J, Blanchet FG, Friendly M, Kindt R, Legendre P, McGlinn D, et al. vegan: Community Ecology Package [Internet]. 2020. [cited 2021 May 6]. Available from: https://CRAN.R-project.org/package=vegan. Accessed 6 May 2021.

[78] R Core Team. R: a language and environment for statistical computing [Internet]. Vienna: R Foundation for Statistical Computing. Available from: https://www.R-project.org. Accessed 6 May 2021.

[79] Bates D, Mächler M, Bolker B, Walker S (2015). Fitting linear mixed-effects models using lme4. J Stat Softw.

