# Supplementary material for: Utilizing a reductionist model to study host-microbe interactions in intestinal inflammation
Source: Microbiome. 2021 Nov 3;9:215. doi: 10.1186/s40168-021-01161-3 (PMC8565002; doi:10.1186/s40168-021-01161-3)

Supplemental Figure 1

A.

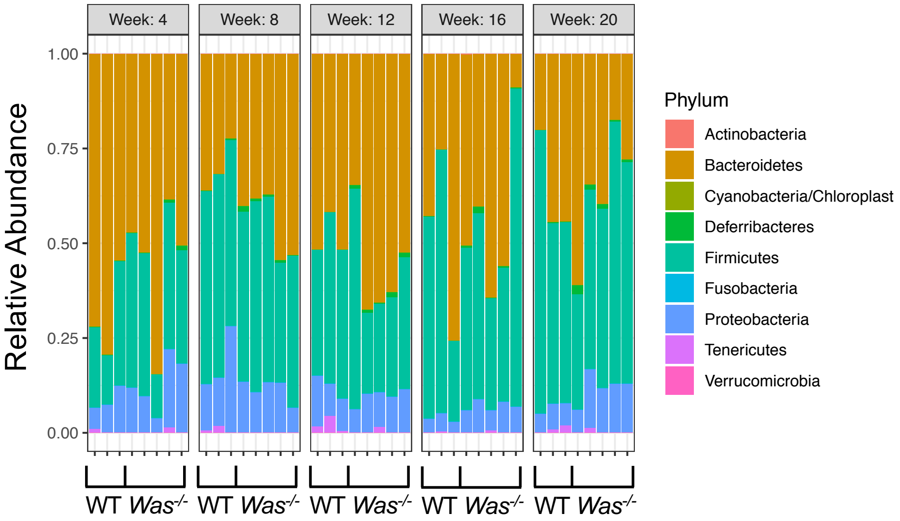

B.

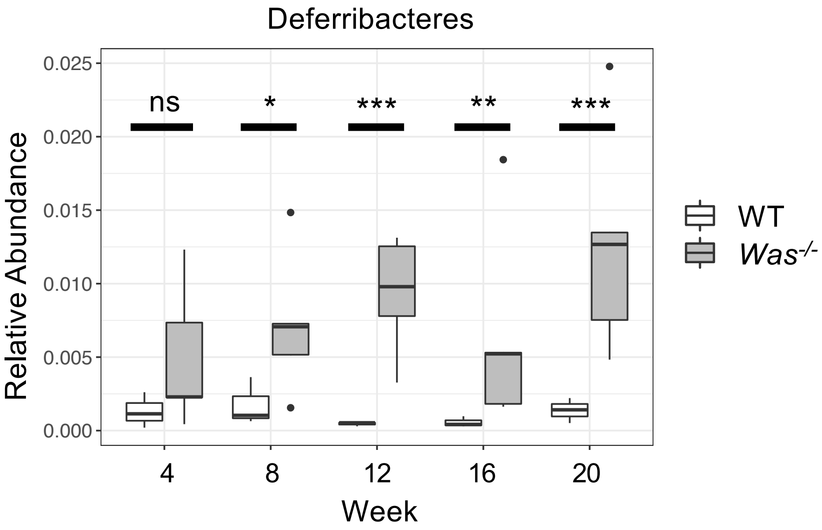

Supplemental Figure 2

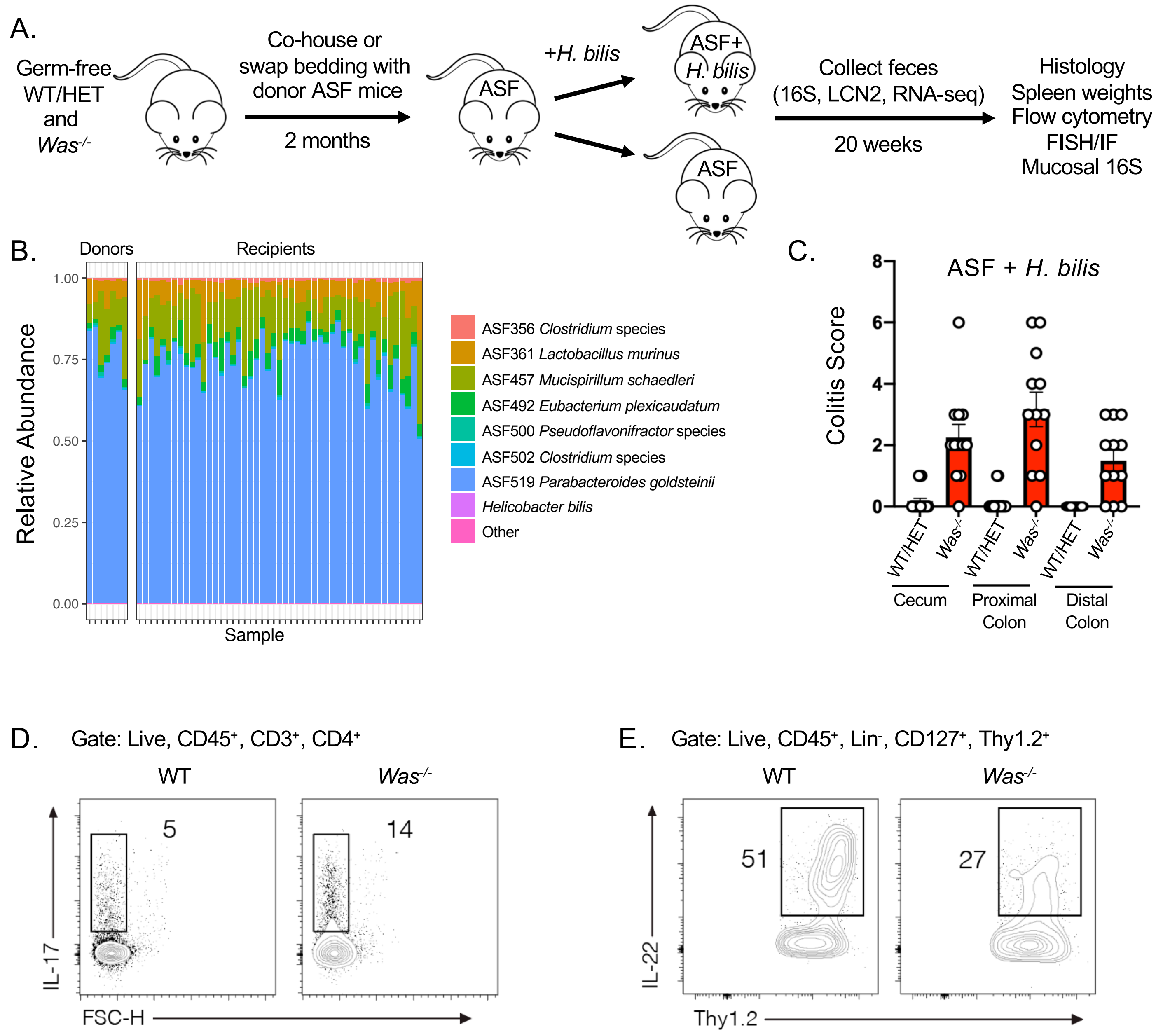

Supplemental Figure 3

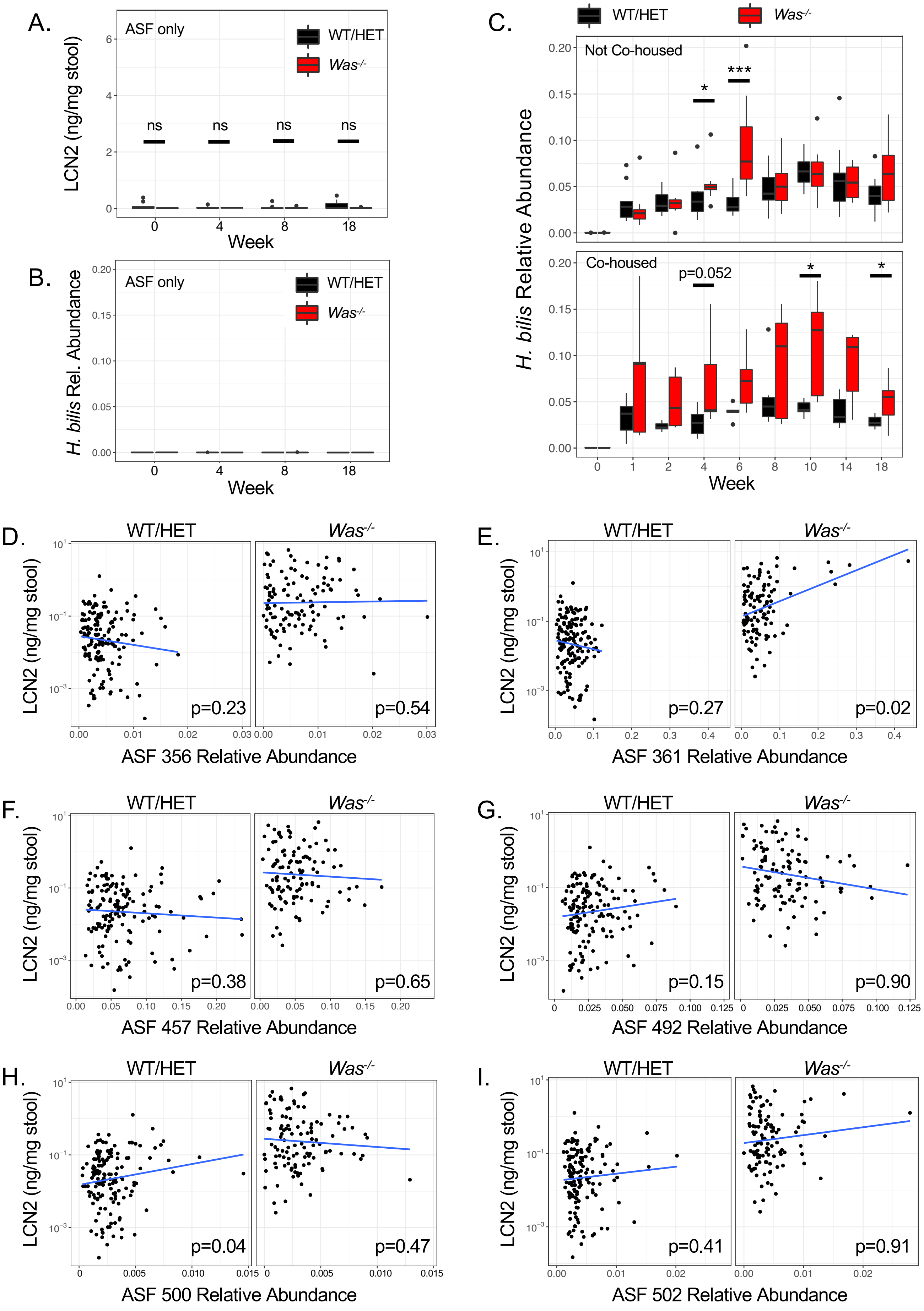

Supplemental Figure 4

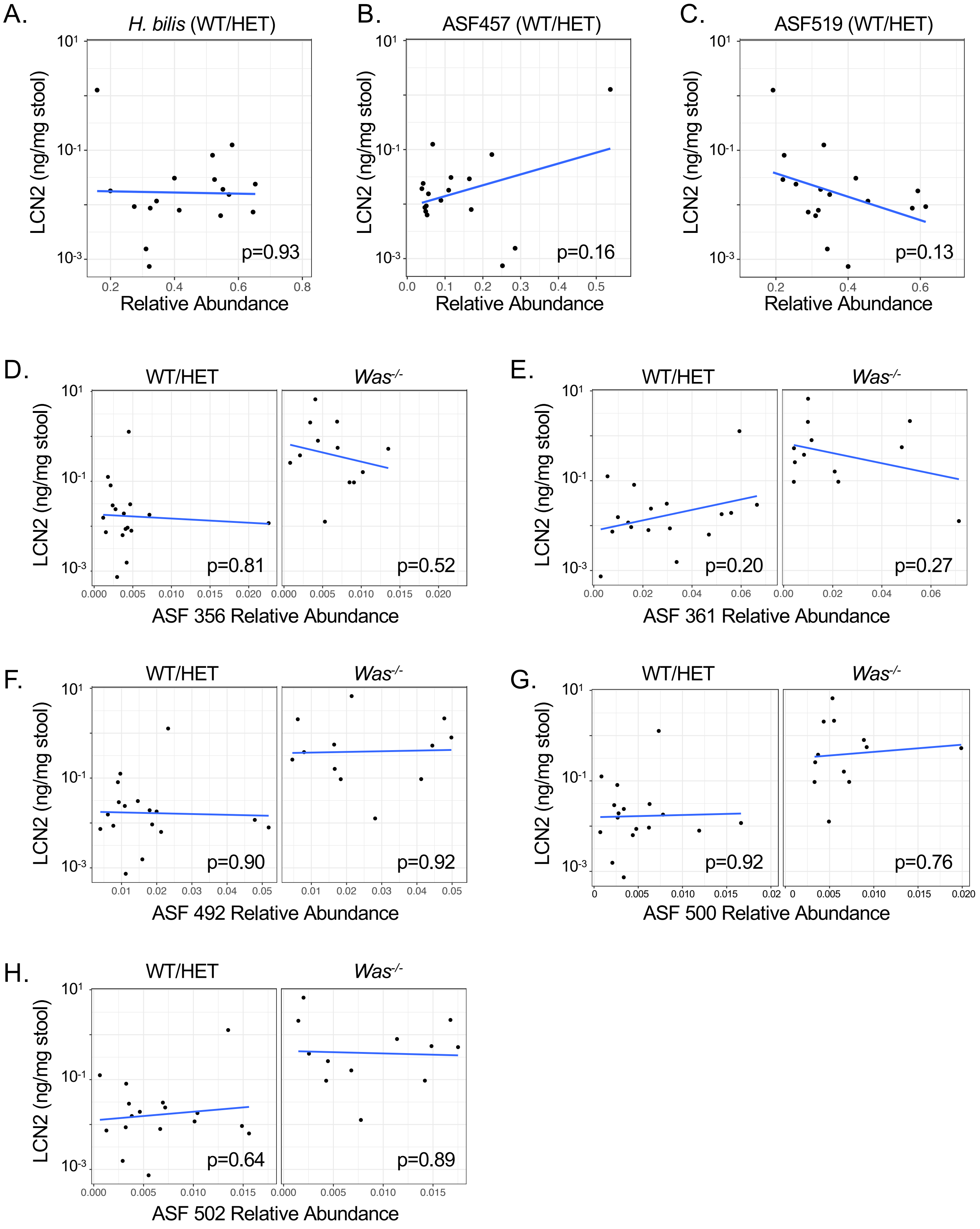

Supplemental Figure 5

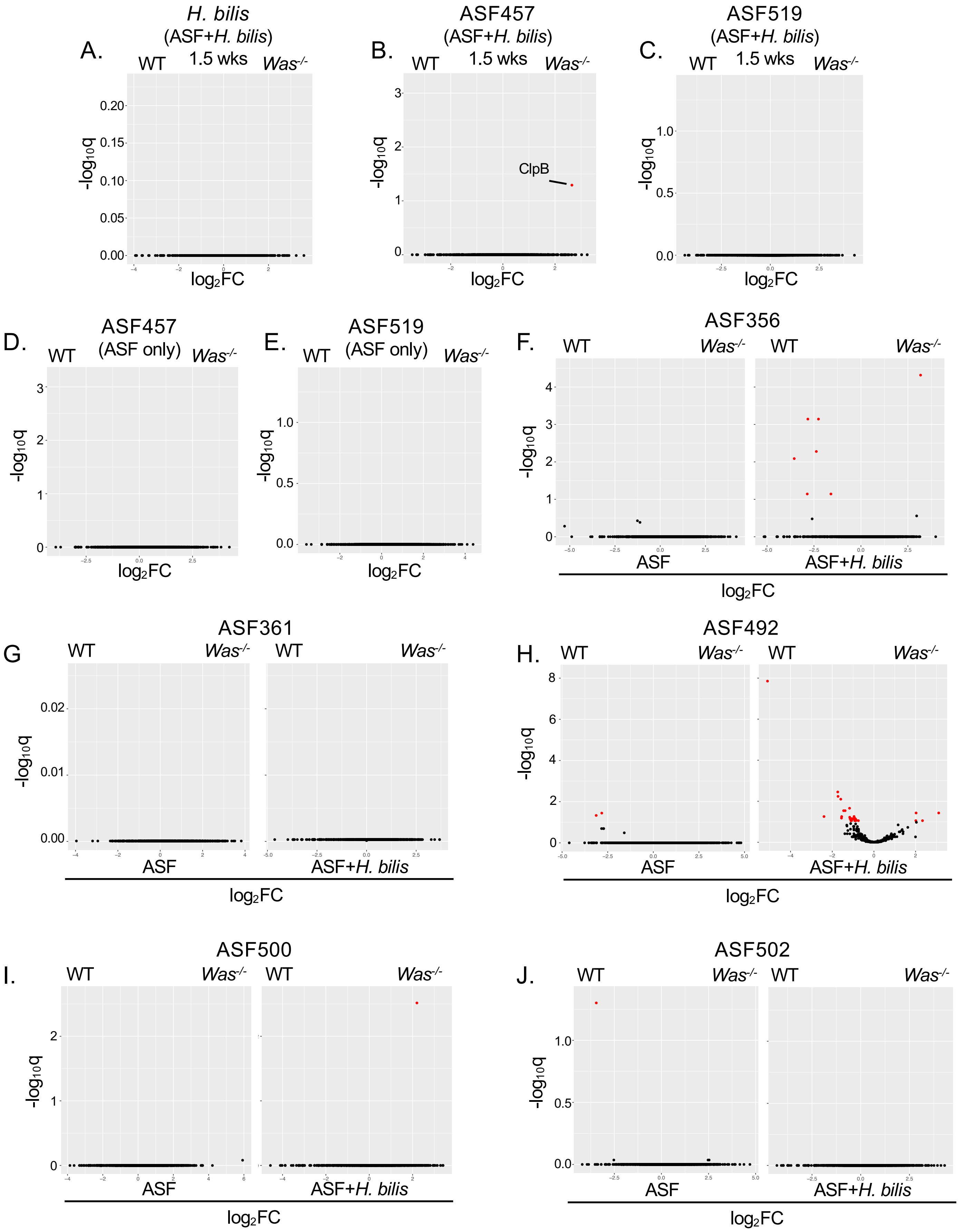

Supplement: Supplementary file 2 — Additional file 1: Fig. S1. WASP deficiency results in altered composition of the fecal microbiota. Fecal microbial composition of Was-/- (n=5) and WT (n=3) mice raised under SPF conditions with weekly bedding exchanges was analyzed monthly between 4 and 20 weeks of age by 16S rRNA gene sequencing. (A) Microbial relative abundances at the phylum level. (B) Relative abundance of the phylum Deferribacteres at each timepoint by genotype. Statistics performed using the DESeq2 R package and adjusted for multiple taxa comparisons. * p < 0.05, ** p < 0.01, *** p < 0.001. Fig. S2. Establishing a reductionist model to study the role of the microbiota in the development of intestinal inflammation. (A) Experimental design. (B) Fecal microbial composition of donor mice harboring the ASF consortium and recipient ex-germ-free mice after 2 months of co-housing (females) or bedding exchanges (males) with donors. Composition assessed based on 16S rRNA sequencing. (C) Quantitative histological colitis scores 20 weeks after gavage with H. bilis in the cecum, proximal colon, and distal colon. (D) Gating strategy for IL-17A+ CD4 T cells. (E) Gating strategy for IL-22+ ILC3s. Lin includes CD3, CD19, CD11b, CD11c, NK1.1, Ly6C, Ly6G. Fig. S3. Correlations between intestinal inflammation and fecal microbial composition. (A-B) Germ free WT/HET (n=11) and Was-/- (n=8) mice colonized with the ASF community but not gavaged with H. bilis served as a control group. Fecal LCN2 (A) and absence of H. bilis (B) were monitored serially. (C) In mice that received H. bilis, H. bilis relative abundance is shown based on whether the mouse was in a cage that contained both genotypes (co-housed) or only one genotype (not co-housed). (D-I) Correlations between log-transformed fecal LCN2 and relative abundances of the indicated ASF members in mice of the indicated genotype colonized with ASF and H. bilis for all timepoints. Tests for linear dependence of log-transformed LCN2 on the relative abundance of each [file 40168_2021_1161_MOESM2_ESM.pdf]
